# Moxifloxacin rescues SMA phenotypes in patient-derived cells and animal model

**DOI:** 10.1007/s00018-022-04450-8

**Published:** 2022-07-22

**Authors:** Camille Januel, Giovanna Menduti, Kamel Mamchaoui, Cecile Martinat, Ruben Artero, Piotr Konieczny, Marina Boido

**Affiliations:** 1 INSERM/UEVE, UMR 861, Université Paris Saclay, I-STEM, AFM-Telethon, Rue Henri Desbruères, 91100 Corbeil-Essonnes, France; 2 Department of Neuroscience “Rita Levi Montalcini”, Neuroscience Institute Cavalieri Ottolenghi, University of Turin, Regione Gonzole 10, Orbassano, 10043 Turin, TO Italy; 3 Sorbonne Université, Inserm, Institut de Myologie, Centre de Recherche en Myologie, 75013 Paris, France; 4 University Institute of Biotechnology and Biomedicine (BIOTECMED), Universitat de València, Street Dr. Moliner, 50, 46100 Burjasot, Valencia Spain; 5 Translational Genomics Group, Incliva Biomedical Research Institute, Avenue Menéndez Pelayo 4 acc, 46010 Valencia, Spain

**Keywords:** Spinal muscular atrophy, Drug repurposing, SMN2 splicing, Motoneurons, SMA delta7 mice

## Abstract

Spinal muscular atrophy (SMA) is a genetic disease resulting in the loss of α-motoneurons followed by muscle atrophy. It is caused by knock-out mutations in the *survival of motor neuron 1* (*SMN1*) gene, which has an unaffected, but due to preferential exon 7 skipping, only partially functional human-specific *SMN2* copy. We previously described a *Drosophila*-based screening of FDA-approved drugs that led us to discover moxifloxacin. We showed its positive effect on the *SMN2* exon 7 splicing in SMA patient-derived skin cells and its ability to increase the SMN protein level. Here, we focus on moxifloxacin's therapeutic potential in additional SMA cellular and animal models. We demonstrate that moxifloxacin rescues the SMA-related molecular and phenotypical defects in muscle cells and motoneurons by improving the *SMN2* splicing. The consequent increase of SMN levels was higher than in case of risdiplam, a potent exon 7 splicing modifier, and exceeded the threshold necessary for a survival improvement. We also demonstrate that daily subcutaneous injections of moxifloxacin in a severe SMA murine model reduces its characteristic neuroinflammation and increases the SMN levels in various tissues, leading to improved motor skills and extended lifespan. We show that moxifloxacin, originally used as an antibiotic, can be potentially repositioned for the SMA treatment.

**Supplementary Information:**

The online version contains supplementary material available at 10.1007/s00018-022-04450-8.

## Introduction

Spinal muscular atrophy (SMA) is a rare genetic neuromuscular disorder caused by the loss of alpha-motoneurons (MNs) in the spinal cord and brainstem nuclei with an incidence of 1 in 6000–10,000 children. Five clinical types of SMA have been described (0, I, II, III, and IV), ranging from the complete absence of motor function and infant mortality to minor defects with no significant reduction in lifespan. SMA types originate from varying levels of the Survival Motor Neuron protein (SMN), from very low in severe cases to higher levels in the milder forms [reviewed in [1]]. The SMN protein decrease mostly affects MNs, although SMA patients also develop defects in non-CNS tissues, e.g., lungs, heart, skeletal muscles, or peripheral nervous system [reviewed in [2]]. The analysis of samples from human fetuses and data collected from animal models indicate that the muscle growth impairment occurs before the MNs degeneration [3, 4], suggesting that a systemic SMN restoration is needed for the highest therapeutic benefit.

SMA is primarily caused by loss of function mutations in the *SMN1* gene. However, due to duplication in the genome, the *SMN* locus contains two inverted copies of *SMN*, called *SMN1* (telomeric) and *SMN2* (centromeric). The difference in the coding regions of both genes is a C-to-T transition at position + 6 of exon 7 (Ex7) in *SMN2* [5]. This change is critical, as it results in different splicing patterns. In *SMN1*, Ex7 is included giving a full-length, functional SMN protein (FL-SMN), whereas the C-to-T transition in *SMN2* causes Ex7 exclusion in the majority of cases, giving a C-terminally truncated SMN protein (Δ7-SMN). A part of the *SMN2* mRNAs includes Ex7 and gives a functional FL-SMN protein [6], although insufficient to fully compensate for the *SMN1* loss. All the SMA patients carry one or more copies of the *SMN2* gene. The number of *SMN2* copies is inversely correlated with the disease severity, because the amount of functional protein translated from the *SMN2* transcript partially compensates for *SMN1* deficiencies [7].

The FDA approved three SMN modifying treatments to restore the functional protein in the last years. One of them is a gene therapy called onasemnogene abeparvovec (Zolgensma™), which is a gene-replacement therapy for the lack of SMN. In addition, two drugs aim to increase the functional SMN protein by boosting the inclusion of *SMN2* Ex7: an antisense oligonucleotide named nusinersen (Spinraza™) and the small molecule drug risdiplam (Evrysdi™) [8]. Despite the remarkable efficiency of these treatments, they also present disadvantages in administration method, patient exclusion criteria, or access due to the therapy's high cost [9–11]. Moreover, the limited data on the long-term efficacy and safety of these innovative therapies makes it crucial to continue to search for alternative, backup treatments for SMA. The repositioning approach drastically shortens the time needed for a drug to become a new therapy for patients [12–14]. Our previous research combined this strategy with a novel *Drosophila*-based screening method and reported moxifloxacin, a fluoroquinolone antibiotic, to be an effective *SMN2* Ex7 splicing booster in SMA patient fibroblasts [15]. This paper investigates moxifloxacin’s effect on the SMA molecular and phenotypical hallmarks in MNs generated from patient-derived induced pluripotent stem cells and a human in vitro model of neuromuscular junctions (NMJs), and in a severe SMA murine model, further reinforcing its therapeutic potential. The well-known profile of this drug makes it an exciting candidate for a clinical trial.

## Materials and methods

### Human induced pluripotent stem cells

Human induced pluripotent stem cell lines (hiPSC) were generated by reprogramming Coriell Biorepository fibroblasts derived from non-affected and SMA affected patients as previously described [16]. Three independent hiPSC clones were generated from each line of healthy fibroblasts GM03814 and C03 as previously described [17]. Similarly, 3 independent clones of hiPSC were generated from each fibroblast lines issues from SMA type I (GM00232) and type II (GM03813) patients (Supplementary Fig. MM1). Informed consents were obtained from all the patients included in this study, complying with the ethical guidelines of the institutions and with the legislation requirements. Experimental protocols were approved by the French minister of health (2019-A02599-48). In addition to these hiPSC lines, an isogenic control line was generated from one SMA hiPSC line (GM03813) as previously reported [18]. All the different hiPSC lines were grown on culture dishes coated with vitronectine (Gibco) and maintained in iPS-Brew XF medium (Miltenyi Biotec) as previously described [17]. Cell passaging was performed manually every 5 days and culture medium was changed every 2 days.

### Generation of spinal motoneurons from hiPSCs

The conversion of hiPSC into spinal motoneurons was performed as previously described [16]. Briefly, hiPSC were dissociated enzymatically using Stem Pro Accutase (ThermoFisher®) during 5 min at 37 °C, 5% CO_2_ and plated in 25 cm^2^ flasks (Dutscher^®^) at 2 million per flask in an induced-motoneuronal medium supplemented with cytokines every 2 days. After 10 days of differentiation, embryoid bodies were dissociated. Between days 10 and 14, MN progenitors are converted into MNs, and motoneuron phenotype was assessed by immunolabeling for Islet1.

### Co-culture of human iPSC-derived spinal MN and human skeletal muscle cells

Human primary and immortalized non-affected and SMA myoblasts were obtained from the MyoLine platform from the Institute of Myology and provided by Vincent Mouly [19]. They were derived from patients and control biopsies provided by MyoBank, affiliated to EuroBioBank with the French ministry agreement ref AC-2019-3502. Skeletal muscle cells were obtained from a quadriceps muscle biopsy of a 5-day-old unaffected infant (CHQ or Ctl) [20], a paravertebral muscle biopsy of an 11-year-old infant patient suffering from SMA (KM432-7PV or SMA type I) Primary cells were isolated from skeletal muscle, expanded and differentiated as described previously [19]. Briefly, primary and immortalized myoblasts were cultured in a growth medium and cryopreserved. The percentage of myoblast cells was estimated by immunolabelling for Desmin. For co-culture, spinal MNs must be differentiated from hiPSC as described previously, but the embryoid bodies are dissociated at day 14 of differentiation and frozen. 384 micropatterned-well plates were designed and provided by CYTOO SA [21]. For the co-culture the myoblasts were plated at a density of 5,500 cells per well in myoblast growth medium (DMEM/F12-glutamax supplemented with 20% fetal bovine serum (Sigma-Aldrich) and 0.1% penicillin–streptomycin (ThermoFisher) with Y-27632 (10 μM, Stemcell). After 2 days, the growth medium was replaced by the differentiation medium (DMEM/F12-glutamax supplemented with 2% horse serum (ThermoFisher)). On day 4, the hiPSC-derived MNs were thawed and plated at a density of 8,000 cells per well in N2B27 medium supplemented by brain-derived neurotrophic factor (BDNF, 10 ng/mL, Peprotech), glial-derived neurotrophic factor (GDNF, 10 ng/mL, Peprotech), *N*-[*N*-(3,5-Difluorophenacetyl)-l-alanyl]-*S*-phenylglycine t-butyl ester (DAPT, 10 µM, Tocris) and Y-27632 (10 μM, Stemcell). Cultures have been maintained up to 11 days which correspond to 4 days of myogenic differentiation and 7 days of co-culture with hiPSC-derived MNs.

### Treatment of motor neuron cultures with compounds

To analyze the effect of compounds, MN progenitors were plated into poly-l-ornithine/laminin-treated 384-well plates at 3,000 cells per well in N2B27 medium without growth factors. After 4 days of differentiation, “early” spinal MNs were treated with the different compounds using the Bravo Automated Liquid Handling Platform (Agilent Technologies). Cells were treated every 3 days for the next 10 days. A solution of 0.1% DMSO (MilliporeSigma) was used as a negative control. After 10 days, the cell viability was quantified. Moxifloxacin (Cat. No.: HY-66011), risdiplam (Cat. No.: HY-109101) were provided by MedChemExpress, while kenpaullone (Cat. No.: 1398) was provided by Tocris.

### Immunostaining

After fixation with 4% paraformaldehyde (PFA) (Electron Microscopy Sciences) for 7 min at room temperature, cells were incubated overnight at 4 °C with primary antibodies (listed in Table 1). The cells were washed 3 times in PBS buffer and incubated for 1 h at room temperature with appropriate Alexa fluorescent-labeled secondary antibodies (Invitrogen, 1:1,000) and Hoechst (5 μg/mL).

**Table 1:** List of primary and secondary antibodies used in immunofluorescence and immunocytochemistry experiments

Model	Antibodies	Designation	Reference	Host	Provider	Dilution
Motoneurons	Primary	Islet1	AF1837	Goat	R&D systems	1:500
		Tubulin β III (Tuj1)	801201	Mouse	Biolegend	1:1000
		Tubulin β III (Tuj1)	PRB-435P	Rabbit	Biolegend	1:1000
		Myosin Heavy Chain (MF20)	MF20	Mouse	DSHB	1:200
		Acetylocholine receptor (AchR)	mAB 35	Rat	DSHB	1:350
		SMN	Sc-32313	Mouse	Santa Cruz Biotechnology	1:200
	Secondary	Donkey anti-mouse IgG (H + L) alexa fluor 488	A-21202		Thermo Fisher Scientific	1:1000
		Donkey anti-mouse IgG (H + L) alexa fluor 647	A-31571		Thermo Fisher Scientific	1:1000
		Donkey anti-goat IgG (H + L) alexa fluor 568	A-11057		Thermo Fisher Scientific	1:1000
		Donkey anti-rabbit IgG (H + L) alexa fluor 647	A-31573		Thermo Fisher Scientific	1:1000
		Donkey anti-rat IgG (H + L) alexa fluor 488	A-21208		Thermo Fisher Scientific	1:1000
SMA delta 7 mice	Primary	Neurofilament H (NF-H) SMI32	801702	Mouse	BioLegend	1:1000
		Cleaved Caspase 3 (Asp175)	9661	Rabbit	Cell Signaling Technology	1:400
		Glial Fibrillary Acidic Protein (GFAP)	Z0334	Rabbit	DAKO Cytomation	1:500
		Neurofilament 145 kDa Antibody, CT, clone 3H11	MAB1621	Mouse	Sigma-Aldrich	1:100
	Secondary	Cy™2 AffiniPure Donkey Anti-Mouse IgG (H + L)	AB_2340826		Jackson ImmunoResearch	1:400
		Cy™2 AffiniPure Donkey Anti-Rabbit IgG (H + L)	AB_2340612		Jackson ImmunoResearch	1:400
		Cy™3 AffiniPure Donkey Anti-Mouse IgG (H + L)	AB_2340813		Jackson ImmunoResearch	1:400
		Cy™3 AffiniPure Donkey Anti-Rabbit IgG (H + L)	AB_2307443		Jackson ImmunoResearch	1:400

### FastLane quantification of *SMN* mRNA expression

*SMN* mRNA expression from hiPSC-derived MNs treated in 384-well plates was performed using FastLane Cell probe Kit (Qiagen) according to the manufacturer's protocol. Briefly, cells were washed and lysed with Fastlane’s reagents. Without RNA purification step, cell lysates were used directly as templates in real-time one-step reverse transcription PCR using the QuantiTect Probe from FastLane kit combined with specific primers of endogenous control primer set, *18S*, and two *SMN* primer sets, SMN-FL (designed to amplify SMN transcript containing exon 7) and SMN-Δ7 (designed to amplify SMN transcript lacking exon 7). The sequences of the SMN-FL primer set (spanning exons 6, 7, and 8) were forward 5ʹ-CAAAAAGAAGGAAGGTGCTCACATT-3ʹ, reverse 5ʹ-GTGTCATTTAGTGCTGCTCTATGC-3ʹ, and probe 5ʹ-FAM-CAGCATTTCTCCTTAATTTA-NFQ-3ʹ and the sequences of the SMN-Δ7 (spanning the exon 6–8 junction) were forward 5ʹ-CATGGTACATGAGTGGCTATCATACTG-3ʹ, reverse 5ʹ-TGGTGTCATTTAGTGCTGCTCTATG-3ʹ, and probe 5ʹ-FAM-CCAGCAT TTCCATATAATAGC-NFQ-3ʹ. Fold change variations induced by compound treatments were calculated using the 2^−ΔΔ*Ct*^ method with DMSO-treated control cells as reference sample and *18S* housekeeping gene as reference gene.

### HTRF assay to quantify SMN protein expression

SMN protein was also quantified using Homogeneous Time Resolved Fluorescence (HTRF, Cisbio Bioassays) according to the manufacturer’s protocol. This technology allows the detection and quantification of SMN protein in 384 well plate format. For this, cells were lysed in 15 µL of lysate buffer and 10 µL of cell lysate were transferred to a 384-well small volume white plate containing 5 µL of the antibody solution (1:100 dilution of anti-SMN d2 and anti-SMN cryptate) (Cisbio). The plate was incubated overnight at room temperature. Fluorescence was measured at 665 nm and 620 nm on a CLARIOstar microplate reader (BMG Labtech). Ratio values of (665 nm emission/620 nm emission) × 10 000 were used to calculate Δ*F* (%) according to the following equation:

ΔF (%) = [(Sample ratio − Negative control ratio) / Negative control ratio] × 100.

### Image acquisition and analysis

Image acquisitions were performed using the automated imaging Cell Insight CX7 HCS Platform (Cellomics Inc). Masks and algorithms were developed with the HCS Reader software for automated quantification of the percentage of cells stained for ISL1, compared to the total number of cells. Co-culture images were acquired using Spinning Disk microscopy (Zeiss) with the 20X objective and a z-stack plan. For quantification of the AChR clustering and neuritic network, each images taken were processed as a maximum projection and analyzed using Fiji Software (Supplementary Fig. MM2). The total area and mean size of AChR clusters were determined by an algorithm developed in-house. The neurite outgrowth was measured by applying an intensity threshold and a background correction to detect the neurites stained by Tuj1. The number of branches was measured as intersections between neurites from the cluster of soma.

### Animal model and genotyping

To evaluate the effect of moxifloxacin in vivo, we used delta 7 mice (Stock No. 005025; Jackson Lab, Bar Harbor, ME, USA) as a murine model of severe SMA.

Animals had free access to food and water, and were kept into regular cages under 12/12-h light/dark cycle. All efforts were made to minimize the number of animals used and the suffering levels.

The animals were genotyped at P0–1 by PCR assays [22], performed on DNA extracted from the tail, to assess the presence of the two human transgenes (SMN2 and SMN∆7) and the three possible genotypic variants of the Smn locus mice (Smn+/+, Smn+/– or Smn−/−), according to [23].

Data were obtained from tissues harvested from delta 7 mice, sacrificed at P12. An additional group of animals was used for survival analysis. A total of 64 delta 7 SMA mice were used for histological, molecular, behavioral and survival analyses. Moreover, 10 WT mice were used for weight analysis (*n* = 8) and western blot (*n* = 2).

All the in vivo experiments have been carried out by blinded researchers, in a randomized manner.

### Moxifloxacin or vehicle administration

SMA animals were divided into two groups: Vehicle (VHL, 2% DMSO in NaCl 0.9% solution) and moxifloxacin (75 mg/kg; moxifloxacin hydrochloride, BAY 12-8039, MedChemExpress, NJ, USA). The animals were daily injected, subcutaneously, starting from P2 to P12 (or until sacrifice for the animals involved in the survival study) with VHL or moxifloxacin (range of injected volume: 1–48 µl, depending on age and body weight). Additional animals (VHL *n* = 23; moxifloxacin *n* = 12) were sacrificed for humanitarian endpoints if and when they exhibited excessive weight loss (greater than 21 ± 5%) associated with severe neurological/motor symptoms, and were included into survival analysis.

### Weight assessment and behavioural tests

Weight and motor performance assessment was performed daily, from P2/P4 (depending on the test) to P12 on both treated mice groups (VHL *n* = 21; moxifloxacin *n* = 9). Before starting the tests, the mice were moved in a room (with low light or no noise); between the different trials, the pups were maintained on a heated pad (at a controlled temperature of 37 °C) until the end of the tests. All pups were then returned to maternal care after handling.

Delta 7 mice were first weighed and then from P2 underwent the following motor tests: Tail suspension, Righting reflex, Hindlimb suspension, Negative geotaxis (the latter was used from P4), specifically designed for SMA pups (described in detail in [24]). In particular, for the hindlimb suspension test, we only evaluated the hindlimb posture by assigning a score, according to [24].

### Animal sacrifice, tissue collection and processing

For immunoblotting analysis, five animals for each group (VHL *n* = 5, moxifloxacin *n* = 5) were sacrificed by cervical dislocation at age P12: the whole spinal cord and skeletal muscles (quadriceps, gastrocnemius and diaphragm) were harvested, rapidly frozen in liquid nitrogen and then stored at − 80 °C.

For histochemical (Nissl staining) and immunohistochemical analysis, at P12 a number of delta 7 mice (VHL *n* = 4, moxifloxacin *n* = 4) were anesthetized by gaseous anaesthesia (3% isoflurane vaporized in O_2_/N_2_O 50:50) and perfused transcardially with 4% buffered PFA, pH 7.4. Then, spinal cord lumbar tract and skeletal muscles (quadriceps and gastrocnemius) were dissected, postfixed in 4% PFA for 2 h and incubated overnight in 30% sucrose in 0.1 M phosphate buffer solution. Next, samples were embedded and frozen in cryostat medium (Killik, Bio-Optica): the spinal cord samples were cut into transverse 40 µm thick free-floating sections and stored at 20 °C in an antifreeze solution (30% ethylene glycol, 30% glycerol, 10% PB; 189 mM NaH_2_PO_4_; 192.5 mM NaOH; pH 7.4), while the muscle samples were cut longitudinally in 30 µm thick slices, directly on 4% gelatin-coated slides, which were air-dried overnight and then stored at − 20 °C.

For additional histological analysis [haematoxylin/eosin (H/E)], a group of treated delta 7 mice (VHL *n* = 4, moxifloxacin *n* = 4) were sacrificed by cervical dislocation at P12, then quadriceps and gastrocnemius skeletal muscles were harvested, embedded in cryostat medium and frozen (Killik). Skeletal muscles were next cut into transverse 30 µm thick slices, collected directly on 4% gelatin-coated slides, which were air-dried overnight and then stored at − 20 °C.

### Western blot analysis

For the analysis of the expression of SMN protein by western blot (WB), spinal cord and skeletal muscle samples were homogenized on ice using a pestle homogenizer in radioimmunoprecipitation assay buffer (RIPA lysis buffer) (Merck Life Sciences) supplemented with 1 mM PMSF, 1 mM DTT, 2 mM sodium orthovanadate (ThermoFisher Scientific) and 1 × complete™ Protease Inhibitor Cocktail (Merck Life Sciences). Sample homogenates were incubated on ice for 20 min before being centrifuged for the insoluble material separation at 14,000 × *g* for 20 min at 4 °C. Total protein concentration was assayed on supernatants using Bradford reagent (Bio-Rad). Protein denaturation was performed with NuPAGE^®^ LDS Sample Buffer supplemented with NuPAGE^®^ Sample Reducing Agent (ThermoFisher Scientific), by heating at 95 °C for 5 min. SDS–PAGE and transfer were performed on 4–20% Mini-PROTEAN® TGX™ Precast Protein Gels and Trans-Blot^®^ Turbo™ mini nitrocellulose membranes using a Trans-Blot® Turbo™ transfer System (Bio-Rad), respectively. Nonspecific binding sites were blocked using 5% non-fat dried milk in PBS-0.2% Tween-20 (Merck Life Sciences) (PBS-T) for 1 h at room temperature (RT) under shaking. All membranes were incubated overnight at 4 °C under shaking with SMN-antibody solution (diluted 1:2,000 in 2% nonfat dried milk in PBS-T) (mouse–anti-SMN, BD Bioscience 610646) and in Vinculin-antibody solution (diluted 1:2,000 in 2% nonfat dried milk in PBS-T) (Monoclonal mouse Anti-Vinculin, Clone Hvin-1, Merck Life Sciences) as loading control. HRP-conjugated secondary antibody (Goat Anti-Mouse IGG HRP, Bio-Rad) was diluted 1:10,000 in 2% nonfat dried milk in PBS-T and incubated for 1 h at RT under shaking. Immunolabeling was detected with Clarity™ Western ECL Blotting Substrates (Bio-Rad) using the ChemiDoc™ imaging system (Bio-Rad).

For immunoblotting analysis of SMN protein levels in SMA tissues, the densitometric quantitation of bands intensity for the SMN protein levels was calculated with reference to Vinculin protein levels (used as loading control), using the Fiji software (Image J, NIH) (Supplementary Fig. MM3).

### JESS simple western

For the SMN immunodetection in mouse tissues, we used the Jess Simple Western system, an alternative for classical western blotting in which all assay steps from protein separation, immunoprobing, detection, and data analysis are automated. The protein separation was performed for 25 min at 375 V in a commercially available capillary system (one capillary per sample) in which all the classical western blot steps are automatically performed. Next, the total protein was labeled with biotin for 30 min and incubated with a blocking solution (Antibody Diluent) for 5 min. The samples were incubated with SMN antibody (1:10; mouse anti-SMN, BD Bioscience 610646) for 30 min and with secondary antibody (anti-mouse) for another 30 min. The capillaries were washed with RePlex™ solution for 30 min and incubated with Total Protein Streptavidin–HRP solution to detect chemiluminescence signal. All the secondary antibodies and solutions were provided by Bio-techne, and were used according to the manufacturer´s instructions. The data was analyzed and presented using Compass for SW, control and data analysis software for Simple Western instruments. The number of animals on this analysis varied from the immunoblotting experiment due to insufficient amount of some of the samples.

### Stereological motor neuron counting

For the histological examination of spinal cord lumbar tract, 40 µm thick sections were mounted on 4% gelatin-coated slides and Nissl-stained as previously described [30, 36]. The lumbar alpha MNs (tract L3–L5), with an area ≥ 80 μm^2^, were stereologically counted on serial Sects (1 every 320 μm), using the Optical Fractionator technique with the StereoInvestigator software (MicroBrightField Inc.) and a computer-assisted microscope (Nikon Eclipse E600 microscope); then the MN density (reported as MN number/mm^3^) was obtained by the associated data analysis software NeuroExplorer (MicroBrightField). Representative images of spinal lumbar tract sections were acquired with Nikon Eclipse E600 microscope equipped with Optronics MicroFire digital camera.

### Immunofluorescence staining and analysis on spinal cord sections

Spinal cord sections underwent immunofluorescence staining, as previously reported [36]. Sections were incubated overnight with primary antibodies (SMI32, Cleaved Caspase 3 and GFAP) and next with appropriate fluorochrome-conjugated secondary antibodies (primary and secondary antibodies are listed in Table 1), and finally with 4′, 6 Diamino-2 phenyindole Dilactate (DAPI; 1:200; D9564-10MG, Sigma-Aldrich). Samples were washed and coverslips were mounted with anti-fade mounting medium Mowiol. Immunofluorescence analyses were performed with a Leica TCS SP5 confocal laser scanning microscope (Leica Microsystems).

The Cleaved Caspase 3-positive cells were quantified in the ventral horns of the lumbar spinal cord tract (the congruent region for analysis was confirmed by the presence of SMI32-stained MNs) by counting the percentage of Cleaved Caspase 3/number of DAPI-labelled nuclei, using confocal images (40X magnification, 0.5 µm z-step size, 14 µm z-volume, acquisition speed 100 Hz, format 1024 × 1024 pixels). Four animals were analysed for each group, four spinal cord slices were evaluated for each animal.

For the analysis of the astrogliosis, four animals were analysed for each group, six fields for each animal of the spinal cord ventral horns of GFAP-positive cells were acquired by confocal (40X objective, 0.5 µm z-step size, 14 µm z-volume, acquisition speed 100 Hz, format 1024 × 1024 pixels), and then converted in black and white images: the density of immunopositive profiles was quantified using the Fiji software.

### Immunofluorescence staining on skeletal muscle sections and NMJ analysis

After rapid rehydration in PBS, skeletal muscle sections on 4% gelatin-coated slices were stained for NMJ detection. First, the sections underwent antigen retrieval by sequential incubations with 1% SDS (5 min, RT) and sodium citrate solution heated to 95 °C (pH 6, for 7–10 min). Subsequently, non-specific binding sites were blocked using 4% BSA solution, 4% foetal bovine serum and PBS-0.1% Triton-X for 1:30 h, RT. The sections were incubated overnight with the primary antibody (against Neurofilament 145 kDa, listed in Table 1) diluted in the blocking solution for 24 h at 4 °C. The sections were subsequently incubated with appropriate fluorochrome-conjugated secondary antibody (listed in Table 1) (1 h 30 min, RT) and with α-bungarotoxin (BTX) conjugated with Alexafluor-555 (1:500; 11554187, Invitrogen) diluted in 4% BSA and PBS-0.1% Triton-X (1 h 30 min, RT). Finally, slices were incubated with DAPI in PBS 1X for 3 min and then coverslipped with anti-fade mounting medium. Images were acquired with a Leica TCS SP5 confocal laser scanning microscope (Leica Microsystems).

For the analysis of NMJ area and perforation (30–50 NMJs for animal), confocal stacks of quadriceps and gastrocnemius longitudinal sections (40X objective, 0.7 µm z-step size, 20 µm z-volume, acquisition speed 100 Hz, format 1024 × 1024) were analysed with Fiji (Image J, NIH). For NMJ innervation analysis (at least 50 NMJs for animal), the presence and number of NF-positive fibers reaching each NMJ was evaluated and counted with computer-assisted microscope (Nikon Eclipse E600 microscope) to classify the NMJs as mono-innervated, multi-innervated or denervated.

### Histological analysis of skeletal muscle atrophy

For the morphological analysis of skeletal muscles, the quadriceps and gastrocnemius sections were stained with H/E, as previously described [30, 36]. To morphologically evaluate mean fiber area, perimeter, minimum and maximum Feret's diameter, more than 100 fibers for each animal were drawn with Neurolucida software (MicroBrightField Inc.) and a computer-assisted microscope (Nikon Eclipse E600 microscope). Data were obtained by the associated data analysis software NeuroExplorer (MicroBrightField): we then averaged the means obtained from single animals. Representative images of skeletal muscles fibers were acquired with Zeiss Axioscan Z.1 digital slide scanner microscope (Carl Zeiss AG).

### Statistical analysis

Data are expressed as mean ± standard error of mean (SEM). Statistical analysis was performed using GraphPad Prism 8.0 software (GraphPad software, San Diego, CA, USA). Unpaired two-tailed Student’s *t* test was used to compare data between two groups. One-way analysis of variance (ANOVA) was performed to compare in vitro data among three or more groups, followed by a post-hoc Tukey’s multiple comparison test.

Mice weight and motor behavioural test data were evaluated by mixed-effects model with Geisser–Greenhouse correction, whose P value for the column factor (i.e., the statistical difference between delta 7 mice groups according to treatment with VHL or moxifloxacin) was reported, followed by Sidak’s multiple comparison post hoc test to analyse the difference between delta 7 mice treated groups over time. In addition, in those tests expressed with not normally distributed data (i.e., righting reflex and negative geotaxis test), the contingency table analysis (Fisher’s exact test) was also applied. Moreover, for the body weight, according to [24], data were also represented as a Kaplan–Meier plot, defined as the time from one initiating event (i.e., the birth, P0) to a terminating event: the latter one is considered “the postnatal day when (1) the animal lagged in body weight by two standard deviations from the established normal average body weight gain of the WT controls; and (2) the body weight remained two standard deviations below the average on each subsequent days” until the last observation (P12).

Finally, survival assessment between the groups of treated delta 7 mice was performed using the Kaplan–Meier test (censoring the mice that were sacrificed at P12), with Logrank (Mantel–Cox) as a post hoc test, suggested as being more sensitive to differences at the end of the curv﻿e [24].

Values of *p* < 0.05 were considered statistically significant (*p < 0.05; **p < 0.005; ***p < 0.0005; ****p < 0.0001).

## Results

### SMA motoneurons

**Fig. 1:**
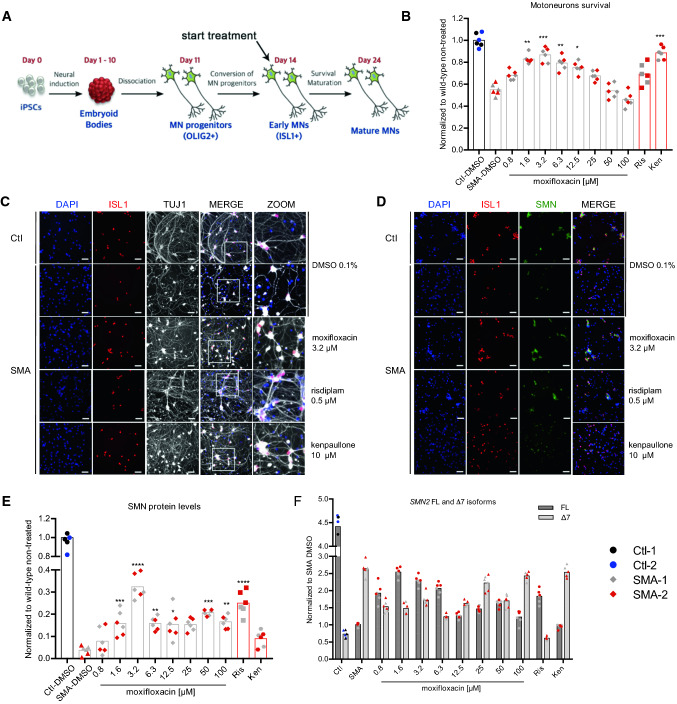
Moxifloxacin increases the SMA motoneurons survival rate. **A** Schematic representation of experimental procedures on SMA and healthy motoneurons derived from induced pluripotent stem cells. **B** MNs survival rate analysis after treatment with moxifloxacin for 10 days. **C**, **D** Representative images of MNs stained using a MN differentiation marker (ISL1; red), cell nuclei (DAPI; blue) and a neural marker (TUJ1; grey) (C) or SMN protein (SMN; green) (**D**) after treatment with moxifloxacin 3.2 μM, kenpaullone 10 μM, risdiplam 0.5 μM, and solvent-treated wild type and SMA controls (DMSO 0.1%). **E** SMN protein levels normalized to the wild-type cells. **F** Quantitative analysis of SMN2-FL and Δ7 isoforms after treatment. Data normalized to SMA cells treated with DMSO (0.1%). In all experiments kenpaullone (neuroprotective drug) at 10 μM and risdiplam at 0.5 μM were used as positive controls. Data represent the mean values ± SD from 3 independent experiments using 2 independent controls (C03 in black and GM0314 in blue), 1 SMA-type I (grey) and 1 SMA-type II (red) cell lines. Statistics were calculated using an ordinary One-Way ANOVA, Tukey’s multiple comparisons test (*p* > 0.05, ns: not significant, *p < 0.05, **p < 0.01, ***p < 0.001, ****p < 0.0001)

We first sought to evaluate the potential effect of moxifloxacin on motoneurons, the primary cell type affected in SMA. The effect of moxifloxacin was evaluated on the survival of MNs derived from induced pluripotent stem cells (iPSCs) from SMA type I and type II patients. The conversion of the human iPSCs into spinal MNs was performed as previously described [16]. Briefly, the protocol used is a four-stage process that involves neural induction, generation of motoneuron progenitors, their conversion into motoneurons (day 14), and their final maturation (day 24) (Fig. 1A). The percentage of ISL1 positive cells was measured at day 14 and compared to the control wild-type MNs (Supplementary Fig. 1). We started the treatment with moxifloxacin at this early stage and continued for the next 10 days until day 24, when a significant decline of the cells' survival is already observed in the SMA condition (Fig. 1B).

Early-born MNs were treated with moxifloxacin at 10 concentrations ranging from 100 to 0.8 µM with the solvent (DMSO) at 0.1%. The FDA-approved molecule risdiplam at 0.5 µM, as well as a neuroprotective JNK inhibitor, kenpaullone were used as a positive control in this assay and also healthy wild-type MNs cultures. The motoneurons survival rate was measured by counting ISL1 positive cells from day 14 to day 24. We observed that moxifloxacin significantly decreased the death rate of the SMA MNs, at levels similar to the healthy cells (Fig. 1B, C). The most effective concentration was 3.2 µM, which is over 150 × lower than the one used previously in SMA fibroblasts [15], showing the high efficiency of the drug in MNs. As previously described, we observed a reduction in the number of motoneurons as detected by quantifying the number of ISL1 positive cells in SMA compared to wild-type conditions, a phenotype that can be normalized after moxifloxacin treatment. Similar to the survival experiment, moxifloxacin at 3.2 µM proved to be the most efficient in increasing the SMN protein levels (Fig. 1D, E). The SMN levels after moxifloxacin treatment are lower than the SMN wild-type levels; nevertheless, the survival rates of wild-type and treated cells are very similar. Finally, to confirm that the increase of the SMN protein in MNs is due to the *SMN2* splicing modulation, we measured the FL and Δ7 isoforms by RT-qPCR. The dose-dependent increase in SMN levels after moxifloxacin treatment reflects the increase of FL isoform levels with simultaneous decrease of Δ7 transcripts (Fig. 1F). Taken together, these results show that moxifloxacin acts as a splicing modifier, promoting the inclusion of *SMN2* Ex7 as it was previously showed in SMA fibroblasts in a dose-dependent manner [15].

### SMA type I myoblasts and motoneurons co-culture

**Fig. 2:**
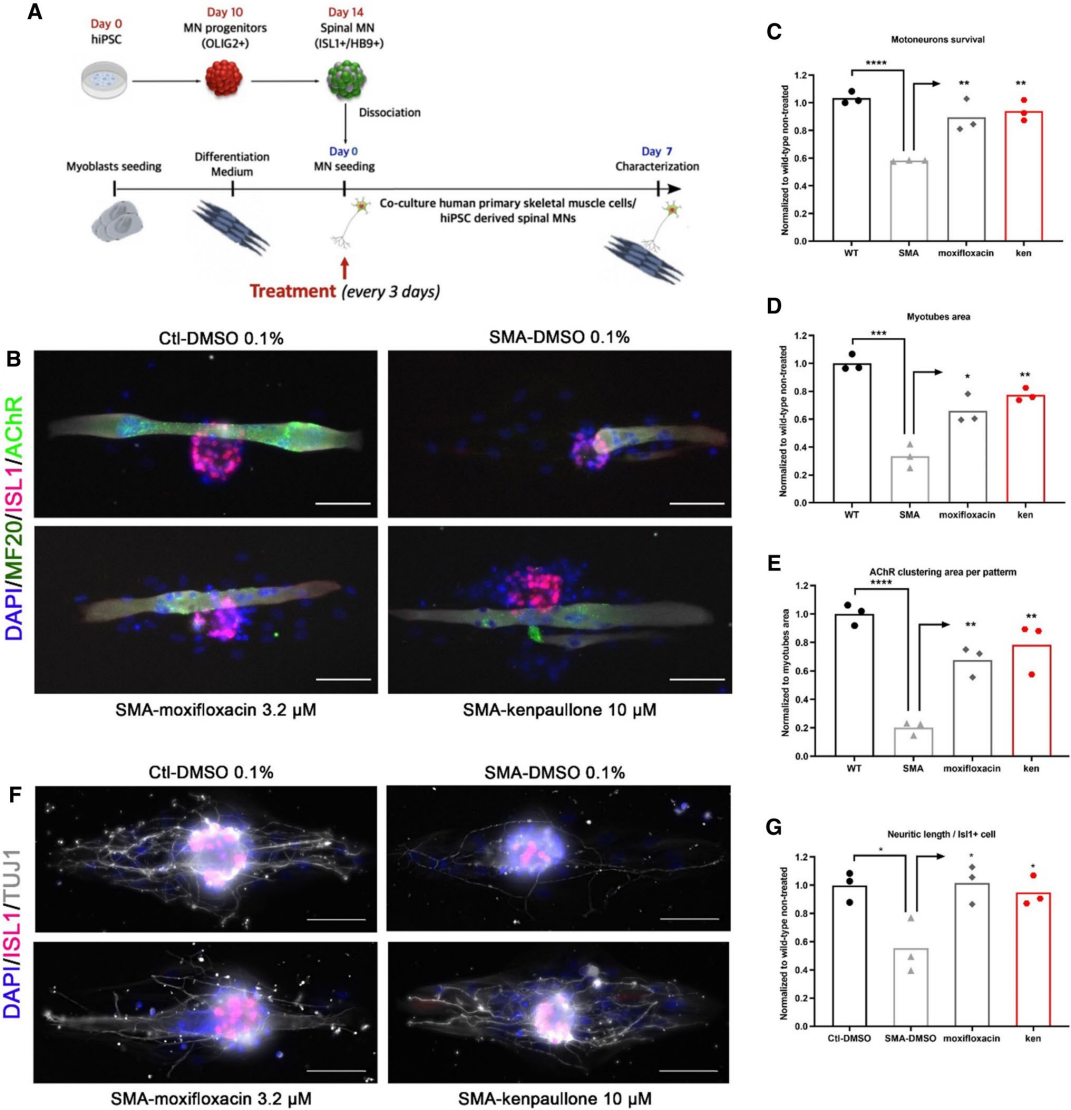
Moxifloxacin increases the SMA myotube area and neuritic length in co-culture with SMA motoneurons. **A** Schematic representation of experimental procedures on co-culture of SMA and healthy conditions. SMA type I or healthy motoneurons derived from induced pluripotent stem cells were co-cultured with SMA type I or healthy myoblasts differentiated into myotubes. **B** Representative images of co-culture treated with moxifloxacin at 3.2 μM, compared to solvent-treated wild-type and SMA cells. MNs were detected with ISL1 (red) and myotubes were marked using MF20 marker (dark green). At the post-synaptic level, acetylcholine receptor was marked with AChR (light green) and cell nuclei were stained with DAPI (blue). Kenpaullone at 10 μM was used as a positive control. **C** MNs survival rate analysis after moxifloxacin treatment compared to non-treated controls. **D** Analysis of SMA myotubes area compared to the non-treated controls. **E** Analysis of acetylcholine receptor clusters after treatment with moxifloxacin compared to non-treated cells. **F** Representative images of co-culture treated with moxifloxacin at 3.2 μM, compared to solvent-treated wild-type and SMA cells. Cocultures were stained with ISL1 (red) and neural marker (TUJ1; grey) and cell nuclei were stained with DAPI (blue). Kenpaullone at 10 μM was used as a positive control. **G** Neuritic length per hiPSC-derived MN was determined after moxifloxacin (3.2 μM) or kenpaullone (10 µM) treatment compared to non-treated control and SMA. Data represent the mean values ± SD from 3 independent experiments using Control and SMA Type I co-culture. Statistics were calculated using an ordinary One-Way ANOVA, Tukey’s multiple comparisons test (*p* > 0.05, ns: not significant, *p < 0.05, **p < 0.01, ***p < 0.001, ****p < 0.0001)

As dramatic alterations at the NMJs have been described prior to symptom onset [25], we next sought to evaluate the therapeutic potential of moxifloxacin in a human in vitro model of NMJ. For this purpose, we used co-cultures between SMA type I hiPSC-derived MNs (and WT MNs as control) and human primary skeletal muscle cells isolated from SMA type I or control patients. To improve myogenic maturation, skeletal muscle cells were grown on micropatterned 384 well plates in which each well contains around 50 micropatterns [21] (Fig. 2A). Co-cultures were treated every 3 days for 1 week, and then different parameters were evaluated, such as the survival of MNs, the myogenic differentiation into myotubes and their ability to connect each other (Fig. 2B).

We observed that in the co-culture conditions, treatment with moxifloxacin led to the normalization of MNs survival as observed in cultures of MNs alone (Fig. 2C), which was also clearly capable of increasing the myotube area (Fig. 2D), restoring the AChR clustering area (Fig. 2E) of SMA myotubes and the neurite outgrowth defect (Fig. 2F, G). When compared to the vehicle-treated cells, the increase was over 20% of the myotube area per pattern and AChR area per pattern normalized to SMA myotube in co-culture with healthy MNs. Thus, moxifloxacin not only enhanced SMA MN survival but improved their ability to establish functional NMJ with SMA myotubes and promoted their growth to approximately 70% of the ability that normal MNs have to rescue SMA myotube growth in vitro.

### Effects of moxifloxacin treatment on weight and motor performances of delta 7 SMA mice

To investigate the efficacy of moxifloxacin administration in the delta 7 SMA murine model [26], we first assessed whether the treatment could affect SMA-related phenotypic signs and motor performances. For these analyses, moxifloxacin- and vehicle (VHL)-treated pups underwent weight assessment and behavioural tests starting from P2 and were sacrificed at P12 (Supplementary Tables 1, 2).

**Fig. 3:**
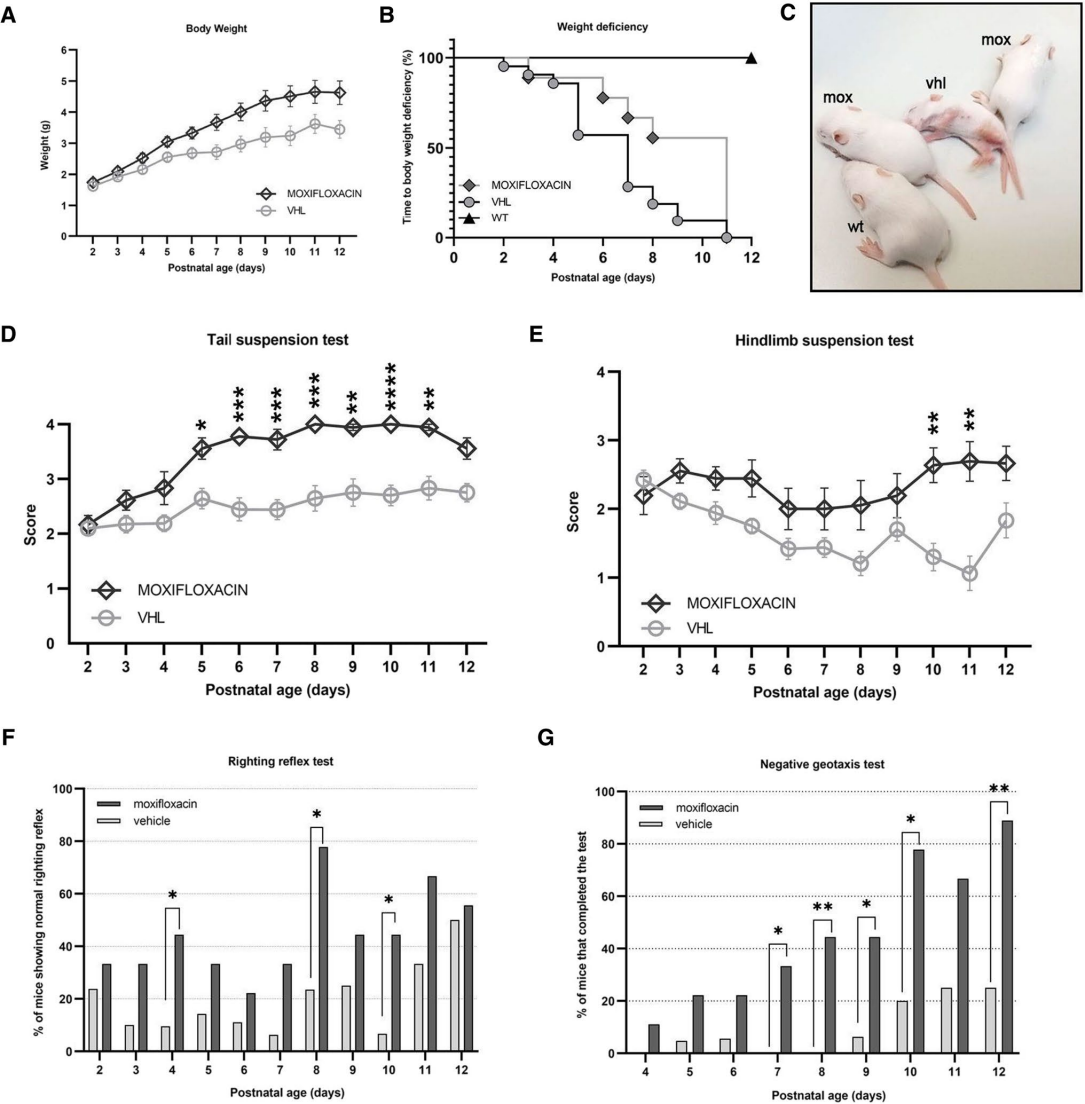
Effect of moxifloxacin on the delta7 mice body mass and locomotor functions. Animals were treated daily from postnatal day 2 (P2) until sacrifice with vehicle (VHL) or moxifloxacin (75 mg/kg) by subcutaneous injections. The animals underwent behavioral motor tests and body weight assessments during this period. **A** Body weight assessment from P2 to P12. Data are expressed as mean ± SEM, VHL *n* = 21, moxifloxacin *n* = 9. Statistical analysis: mixed-effects model with Geisser-Greenhouse correction (*F*_(1,28)_ = 10.38) followed by Sidak’s multiple comparison post hoc test. **B** Event time plot for body weight deviation of VHL, moxifloxacin and WT mice, VHL *n* = 21, moxifloxacin *n* = 9, WT *n* = 8. Statistical analysis: Kaplan–Meier curve test, Log-rank Mantel Cox post hoc test, *P* < 0.05. **C** Comparison of the external appearance of WT, and VHL and moxifloxacin (mox)-treated delta 7 mice in late disease stage. **D** Tail suspension test of treated delta 7 mice. Data are expressed as mean ± SEM, VHL *n* = 21, moxifloxacin *n* = 9. Mixed-effects model with Geisser-Greenhouse correction (F_(1, 28)_ = 34.67) followed by Sidak’s multiple comparison post hoc test. **E** Hindlimb suspension score of treated delta 7 mice. Data are expressed as mean ± SEM, VHL *n* = 21, moxifloxacin *n* = 9. Mixed-effects model with Geisser-Greenhouse correction (*F*_(1, 28)_ = 17.58) followed by Sidak’s multiple comparison post hoc test. **F** Righting reflex of treated delta 7 mice. Data are expressed as mean ± SEM, VHL *n* = 21, moxifloxacin *n* = 9. Statistical analysis: Fisher’s exact test. **G** Negative geotaxis of treated delta 7 mice. Data are expressed as mean ± SEM, VHL *n* = 21, moxifloxacin *n* = 9. Statistical analysis: Fisher’s exact test

The mean body weight (Fig. 3A) showed an overall constant increase in moxifloxacin-treated mice compared to VHL: in particular the statistical analysis showed a significant treatment (*F*_(1,28)_ = 10.38, **p < 0.005) and time (*F*_(2.337,55.85)_ = 51.45, ****p < 0.0001) effects, as well as an interaction between treatment and time (*F*_(10,239)_ = 5.729, ****p < 0.0001). Moreover, as suggested by [24], this kind of representation does not properly shed light on the onset of SMA worsening. Therefore, we converted the 3A graph in a Kaplan–Meier plot: in this way we could observe that VHL mice show an anticipated body weight decrease compared to moxifloxacin-treated SMA mice (Kaplan–Meier curve, Log-rank Mantel Cox test, *p < 0.05), although both groups were clearly worse than WT (used as controls; Fig. 3B, C). Starting from P2/P4, the motor skills of treated mice were evaluated with a battery of behavioural motor tests specifically designed for delta 7 mice pups [24]. The results highlighted a general improvement in hindlimb posture and muscle strength in moxifloxacin-treated mice compared to controls. Indeed, compared to VHL (showing hindlimbs often close together), the treated mice showed higher scores both in the tail suspension test (Fig. 3D) from P5 to P11 and in the hindlimb suspension test (Fig. 3E) with a onefold increase at P10–P11 (P10: + 103%; P11: + 153%), showing almost completely spread hindlimbs.

The results of the righting reflex test (Fig. 3F) showed a positive tendency of moxifloxacin mice in righting themselves when placed on their backs in less time than in control mice, suggesting enhanced muscle strength. Considering the percentage of mice that successfully completed the test, we observed that 44.44%, 77.80% and 44.44% of treated mice righted themselves, respectively, at P4, P8 and P10, compared with 9.52%, 23.53% and 6.67% of controls. In addition, the latency to complete the test is shown in Supplementary Fig. 2A, revealing a statistically significant difference between the groups at P8 (moxifloxacin: 13.23 ± 3.36 s; VHL: 27.84 ± 1.13 s).

Finally, as assessed by the negative geotaxis test (Fig. 3G), vestibular and motor coordination improved significantly in moxifloxacin-treated mice compared to the VHL group, starting from P7 to P12. Interestingly, at P12 the percentage of treated delta 7 mice able to complete the test (in 60 s) was 89% compared to 25% in the control group. The latency to complete the test is shown in Supplementary Fig. 2B and confirms statistically significant differences at P10 and P12 between the two groups.

Overall, these results suggest a general improvement in the health (weight gain), and strength and motor coordination (behavioural tests) of the skeletal muscles of SMA mice upon moxifloxacin administration compared to control animals.

### Moxifloxacin treatment exerts a neuroprotective and anti-inflammatory effect at the level of the murine spinal cord

**Fig. 4:**
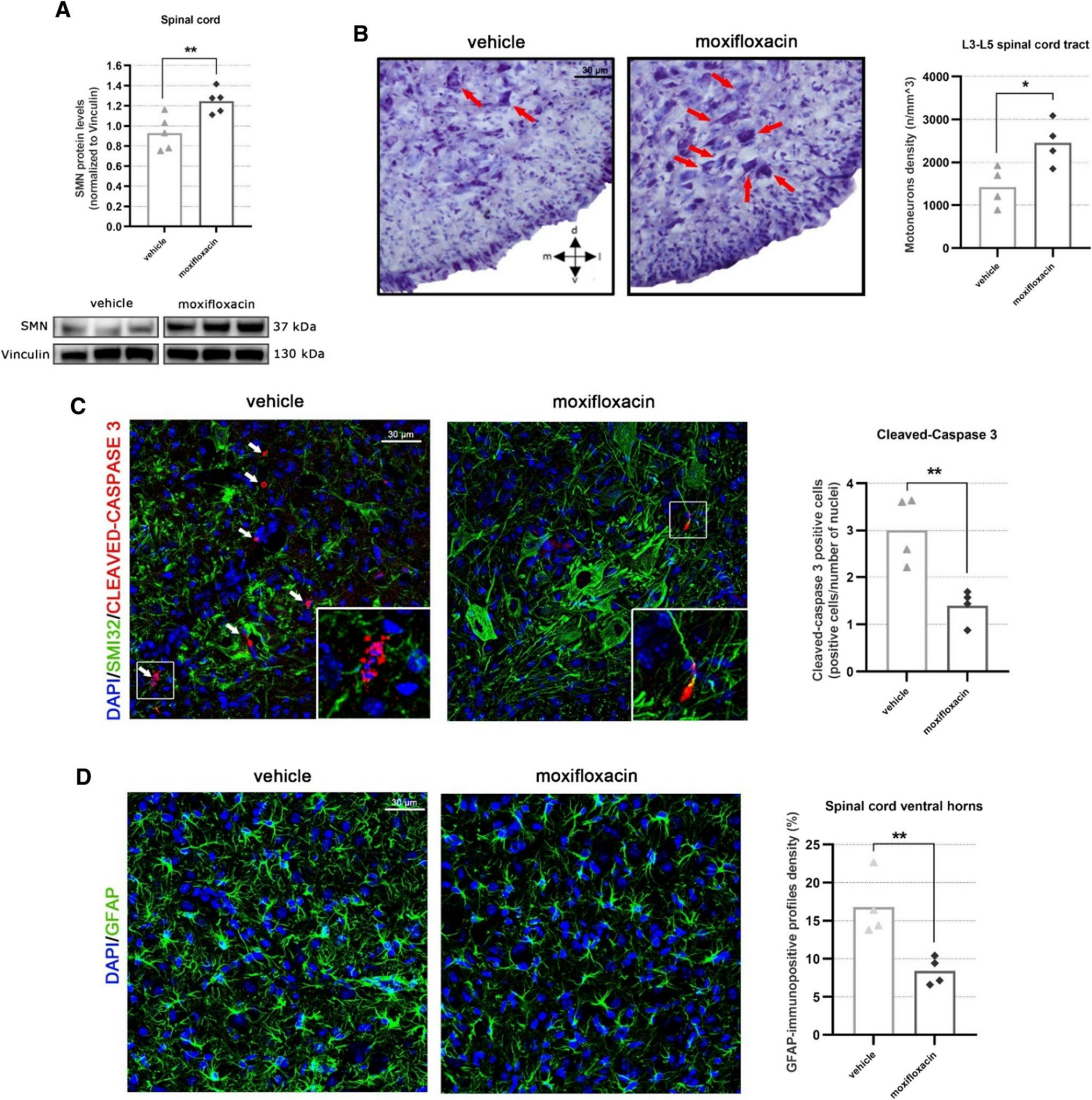
Effect of moxifloxacin on SMN protein levels, MN neurodegeneration and neuroinflammation in the spinal cord of delta 7 mice. **A** Representative immunoblots of SMN protein levels in VHL and moxifloxacin treated delta 7 spinal cord. The intensity of the bands of the SMN protein was calculated with reference to Vinculin protein levels (used as loading control). Data are expressed as mean ± SEM, n = 5 per group, Student’s t-test. **B** Stereological counts of Nissl-stained lumbar alpha MNs in the spinal cord of VHL and moxifloxacin delta 7 mice. Representative images of spinal lumbar tract sections are shown. The lumbar alpha MNs (tract L3-L5), with an area ≥ 10 μm^2^, were stereologically counted and the MN density (reported as MN number/mm3) was assessed. Data are expressed as mean ± SEM, *n* = 5 per group, Student’s t-test. **C** Quantification of the percentage of Cleaved Caspase 3-positive cells in the ventral horns of the lumbar spinal cord tract (L1-L3). Representative confocal images showing the ventral horn of the lumbar spinal tract, where the SMI32-positive MNs are located. The percentage of Cleaved Caspase 3-positive cells was calculated using confocal images on the total number of DAPI-labelled nuclei in ventral horns. Data are expressed as mean ± SEM, *n* = 4 per group, Student’s t-test. **D** Evaluation of reactive astrogliosis (GFAP-positive signal) in the ventral horns of the lumbar spinal cord tract (L1–L3), showed in representative confocal images. The semiquantitative analysis of the GFAP-immunopositive profiles density (expressed as a percentage) displays a reduction of the astrogliosis in moxifloxacin delta 7 mice compared to the VHL group. Data are expressed as mean ± SEM, *n* = 4 per group, Student’s t-test

Previous results reported that moxifloxacin, acting as an *SMN2* modifier, can increase SMN protein levels in patients’ fibroblasts [15] and motoneurons. To confirm such findings in vivo, first we performed an immunoblotting analysis on the spinal cord of VHL and drug-treated delta 7 SMA mice. The SMN protein levels were significantly increased in the moxifloxacin group, by approximately 34% compared with controls (Fig. 4A). This observation was confirmed using a fully automated Simple Western™ platform, which allowed a precise measurement of the SMN protein and normalization to the total protein instead of an endogenous control-like vinculin. With this alternative method, we observed a 57% increase of SMN after the treatment with moxifloxacin (Supplementary Fig. 3A).

Given the observed motor improvements in delta 7 mice and the SMN expression increase in the spinal cord, we then investigated the neuroprotective effects of the drug treatment by evaluating the lower MN survival in the disease model. With this aim, stereological MN counts and quantification of apoptotic profiles in the moxifloxacin and VHL treated mice were performed, analysing the lumbar (L3–L5) spinal cord tract. Our results showed a significantly higher MN density in moxifloxacin-treated mice (2454 ± 261.8 MNs/mm^3^) compared to VHL (1421 ± 233.7 MNs/mm^3^) (Fig. 4B), suggesting that the treatment was effective in delaying the SMA-associated cell death of MNs.

Concerning apoptosis marker quantification (Fig. 4C), by confocal immunofluorescence analysis, we evaluated in experimental and control groups the Cleaved Caspase 3 positive cell percentage in the spinal cord ventral horns (lumbar tract) of delta 7 mice, where the SMI32-positive MNs are located [27]. Results show a significant general reduction of Cleaved Caspase 3 positive cells in moxifloxacin mice (1.39 ± 0.18%) compared to controls (3.00 ± 0.36%), confirming the efficacy of the treatment in delaying cell death mechanisms in delta 7 mice.

Together with MN degeneration, SMA is characterized by increased neuroinflammation in the spinal cord, as it commonly occurs in many neurodegenerative diseases. In particular, astrocytes´ active role and contribution in influencing the severity of SMA pathology has been suggested, and astrogliosis has been found prominently in end-stage delta 7 mice [28]. To investigate whether moxifloxacin neuroprotective effects could be extended to reducing neuroinflammation, we assessed the levels of astrogliosis (GFAP-positive signal) in the spinal cord ventral horns by confocal immunofluorescence (Fig. 4D). Results showed that the treatment significantly reduced the percentage of GFAP-immunopositive profile density by approximately 48% in delta 7 mice with respect to the control group. The modulation of the astrocyte activation could contribute (or be consequent) to moxifloxacin-dependent MN preservation.

### Moxifloxacin increases the SMN expression in the skeletal muscles and counteracts the muscular atrophy in delta 7 SMA mice

**Fig. 5:**
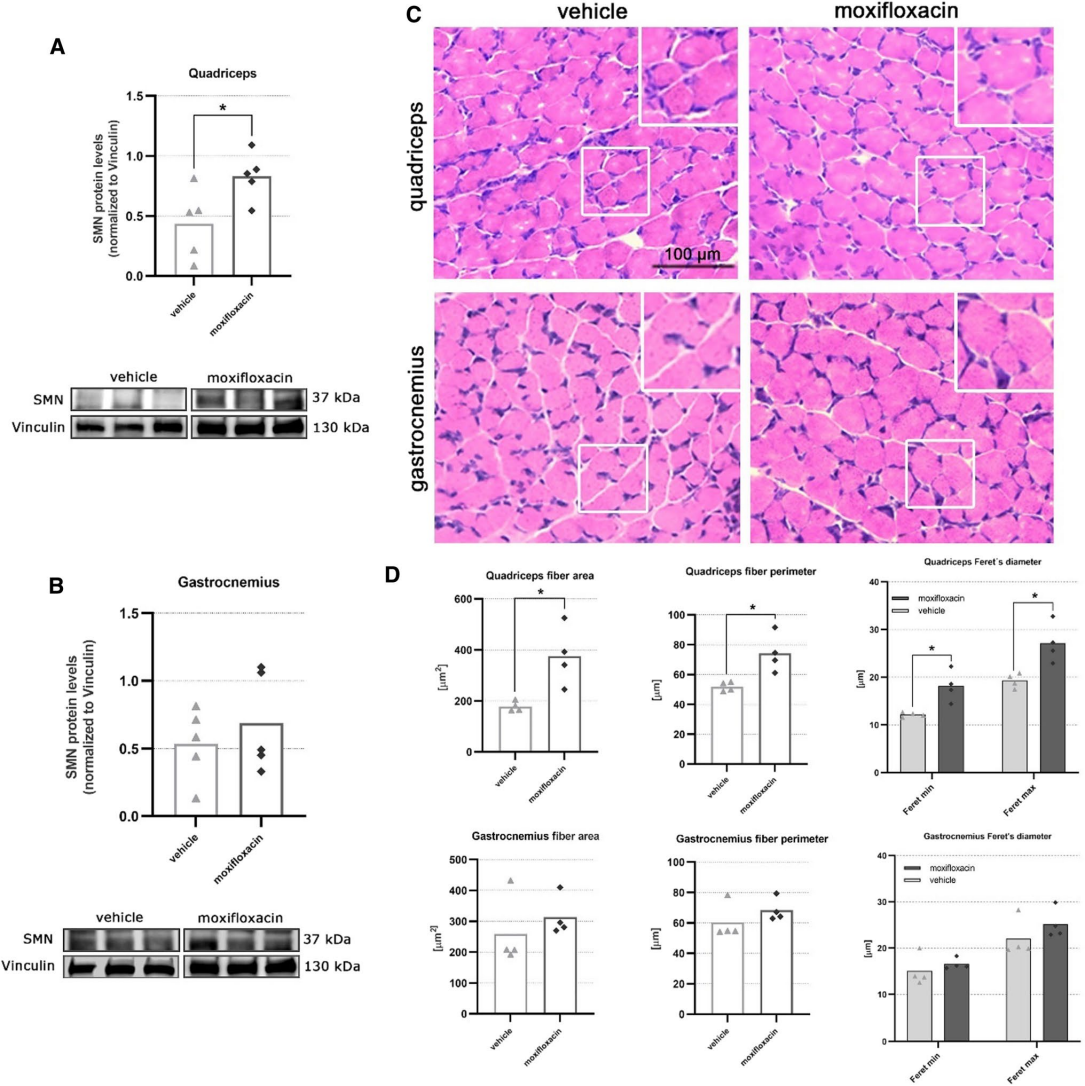
Effect of moxifloxacin on SMN protein levels and muscular fiber morphology in the skeletal muscles of delta 7 mice. Representative immunoblots of SMN protein levels in VHL and moxifloxacin treated quadriceps (**A**) and gastrocnemius (**B**). For each tissue analysis, the intensity of the bands of the SMN protein was calculated by referring to Vinculin protein levels (used as loading control). Data are expressed as mean ± SEM, *n* = 5 per group, Student’s t-test. **C** Comparison of muscular fiber morphology between VHL and moxifloxacin delta 7 mice at P12 of the indicated muscles. Images are representative of H/E-stained muscle sections. **D** For each sample, the measures of the muscle fiber area, the perimeter and the maximum and minimum Feret’s diameter of the muscle fibers of the VHL and moxifloxacin delta 7 mice are reported. At least 100 fibers were analyzed from each animal; the results are shown as the averages obtained from the individual animals. Data are expressed as mean ± SEM, *n* = 4 per group, Student’s t-test

We then verified the therapeutic effect of moxifloxacin at the muscular level by analysing two hindlimb skeletal muscles (quadriceps and gastrocnemius). To evaluate the SMN expression, we performed an immunoblotting analysis on quadriceps and gastrocnemius muscles of VHL and drug-treated delta 7 SMA mice. Significant differences were observed in the quadriceps muscle samples of moxifloxacin mice, which showed an increase in SMN protein of approximately 91% compared to controls (Fig. 5A). While reporting a positive trend of increasing SMN protein levels in moxifloxacin mice (by 28%), the results with the gastrocnemius muscle were not significantly different between the two groups (Fig. 5B). The samples from both muscles were also analysed using the Simple Western™ and in the case of the quadriceps we observed a 78% increase in SMN levels normalized to total protein (Supplementary Fig. 3B). The very low basal SMN levels detected by Simple Western™ in gastrocnemius did not provide sufficient signal to perform a reliable analysis.

Overall, SMN immunodetection results in muscles confirmed the previous findings in the spinal cord, indicating that moxifloxacin treatment can increase SMN protein levels in SMA tissues in the central nervous system and peripheral levels.

Prompted by these positive results, we then verified the efficacy of moxifloxacin on muscle trophism. To this aim, we first performed morphological analyses of the H/E-stained quadriceps and gastrocnemius muscles at P12 (Fig. 5C). We evaluated the mean fiber area, perimeter, and the Feret’s diameters of 100 muscle fibers per animal (Tables 2, 3; Fig. 5D). Concerning the quadriceps, the results displayed that the moxifloxacin-treated mice showed a significant increase in fiber area (+ 100%), fiber perimeter (+ 43%), and Feret's diameters (min Feret: + 49%, Max Feret: + 40%). Regarding the analysis of gastrocnemius morphology, only a slight tendency to higher fiber parameters was evident in delta 7 mice treated with moxifloxacin compared to VHL animals, without reaching a statistically significant difference. These results suggest that moxifloxacin treatment can effectively delay skeletal muscle fiber atrophy and are consistent with the unchanged levels of SMN in gastrocnemius.

**Table 2:** Analysis of quadriceps fiber area, perimeter and Feret’s diameters in VHL and moxifloxacin treated delta 7 mice

	VHL	MOXIFLOXACIN	Student’s t-test
Area (µm^2^)	177.50 ± 9.65	375.9 ± 58.41	*p* = 0.015*
Perimeter (µm)	51.70 ± 1.54	74.17 ± 6.44	*p* = 0.015*
Min Feret’s diameter	12.16 ± 0.23	18.15 ± 1.62	*p* = 0.010*
Max Feret’s diameter	20.94 ± 0.89	30.08 ± 2.78	*p* = 0.020*

(*p < 0.05; **p < 0.005; ***p < 0.0005; ****p < 0.0001)

**Table 3:** Analysis of gastrocnemius fiber area, perimeter and Feret’s diameters in VHL and moxifloxacin treated delta 7 mice

	VHL	MOXIFLOXACIN	Student’s t-test
Area (µm^2^)	259.10 ± 57.54	314.00 ± 32.41	*p* = 0.437
Perimeter (µm)	60.32 ± 5.95	68.35 ± 3.74	*p* = 0.296
Min Feret’s diameter	15.07 ± 1.68	16.56 ± 0.59	*p* = 0.433
Max Feret’s diameter	22.04 ± 2.08	25.20 ± 1.62	*p* = 0.270

(*p < 0.05; **p < 0.005; ***p < 0.0005; ****p < 0.0001)

### Moxifloxacin administration also improves NMJ phenotype in delta 7 SMA mice

Given the positive effects of moxifloxacin on skeletal muscle trophism, NMJ innervation and morphology were also assessed by analysing moxifloxacin- and VHL-treated quadriceps and gastrocnemius muscle samples from delta 7 mice at P12.

**Fig. 6:**
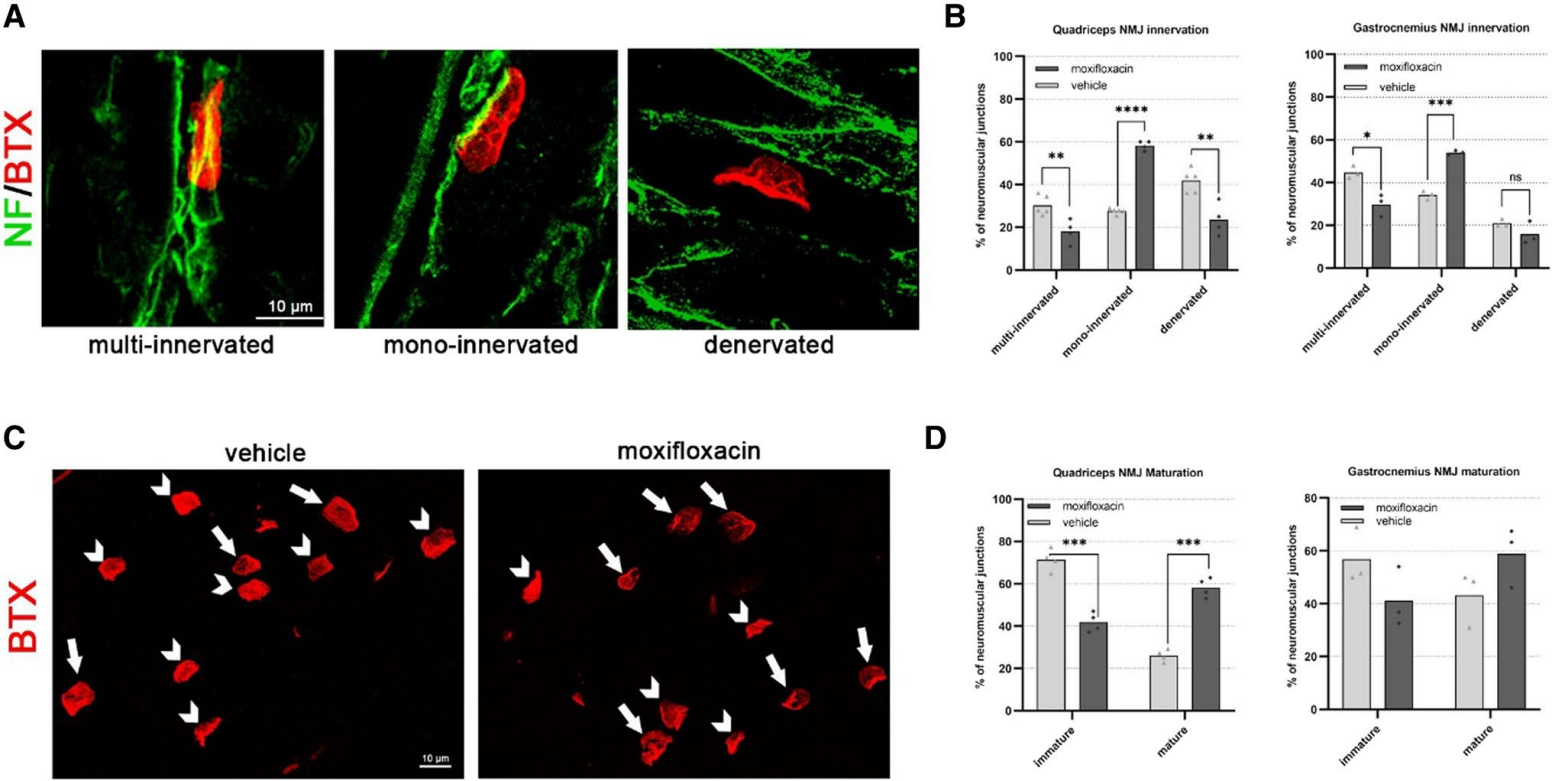
NMJ innervation and maturation comparison between VHL and moxifloxacin delta 7 mice at P12. **A** Double immunostaining against BTX (red) and NF (green) is employed to analyze the plaque innervation: representative confocal images of mono-innervated, multi-innervated, and denervated NMJs are shown. Scale bar = 10 µm. **B** Quantification of the percentage of mono-innervated, multi-innervated, and denervated NMJs in quadriceps and gastrocnemius samples of VHL and moxifloxacin delta 7 mice. **C** Representative confocal images (on the left) showing NMJ maturation, evaluated by BTX-immunostaining: the endplates are classified as immature (no perforations, indicated by triangle arrows) and mature (i.e., showing perforations, indicated by arrows). Scale bar = 10 µm. **D** Percentage of NMJ maturation was evaluated in quadriceps and gastrocnemius of VHL and moxifloxacin delta 7 mice: data are expressed as mean ± SEM, *n* = 4 per group, Student’s t-test: *p < 0.05, **p < 0.01, ***p < 0.005, ****p < 0.001

During development, myofibers undergo a multiple to single innervation process: therefore, the presence of mono-innervated NMJs, together with a high number of endplate perforations, indicates a more mature stage [25]. NMJ innervation was analysed for the number of neurofilament (NF) contacting the endplate (Fig. 6A, B); results showed a significant decrease in multi-innervated NMJs (18.10 ± 2.70%) and an increase in the percentage of mono-innervated junctions in moxifloxacin treated quadriceps (58.31 ± 1.06%), compared to controls (VHL multi-innervated NMJs 30.32 ± 2.07%; VHL mono-innervated NMJs 27.62 ± 0.57%). We also observed a significant reduction in denervated endplates in moxifloxacin mice compared to controls (moxifloxacin: 23.58 ± 3.73%; VHL: 41.87 ± 2.49%). Similarly, NMJ innervation analysis in gastrocnemius showed in moxifloxacin treated mice a significant reduction in multi-innervated NMJ percentage (moxifloxacin: 29.77 ± 2.98%; VHL: 44.77 ± 1.69%) and an increase in mono-innervated NMJ percentage in comparison with the VHL group (moxifloxacin: 54.00 ± 0.57%; VHL: 34.20 ± 1.17%). No significant difference between the two groups was observed in the percentage of denervated NMJs. The presence of perforations in the NMJs was also assessed (Fig. 6C, D), and we classified the endplates as mature (i.e., showing perforations) and immature (no perforations) [29, 30]. The results showed that moxifloxacin treatment significantly increased the percentage of mature NMJs in delta 7 mice compared to the VHL group (moxifloxacin: 58.22 ± 2.26%; VHL: 26.05 ± 1.42%) in the quadriceps. A slight increase in the percentage of mature NMJs following treatment was also observed in the gastrocnemius (moxifloxacin: 58.86 ± 6.54%; VHL: 43.16 ± 6.09%), although without reaching a statistically significant difference.

Therefore, the administration of moxifloxacin to delta 7 mice supports the skeletal muscle trophism and leads to acquiring a more mature phenotype of NMJs and a better muscular innervation.

### Effects of moxifloxacin treatment on delta 7 SMA mice survival

**Fig. 7:**
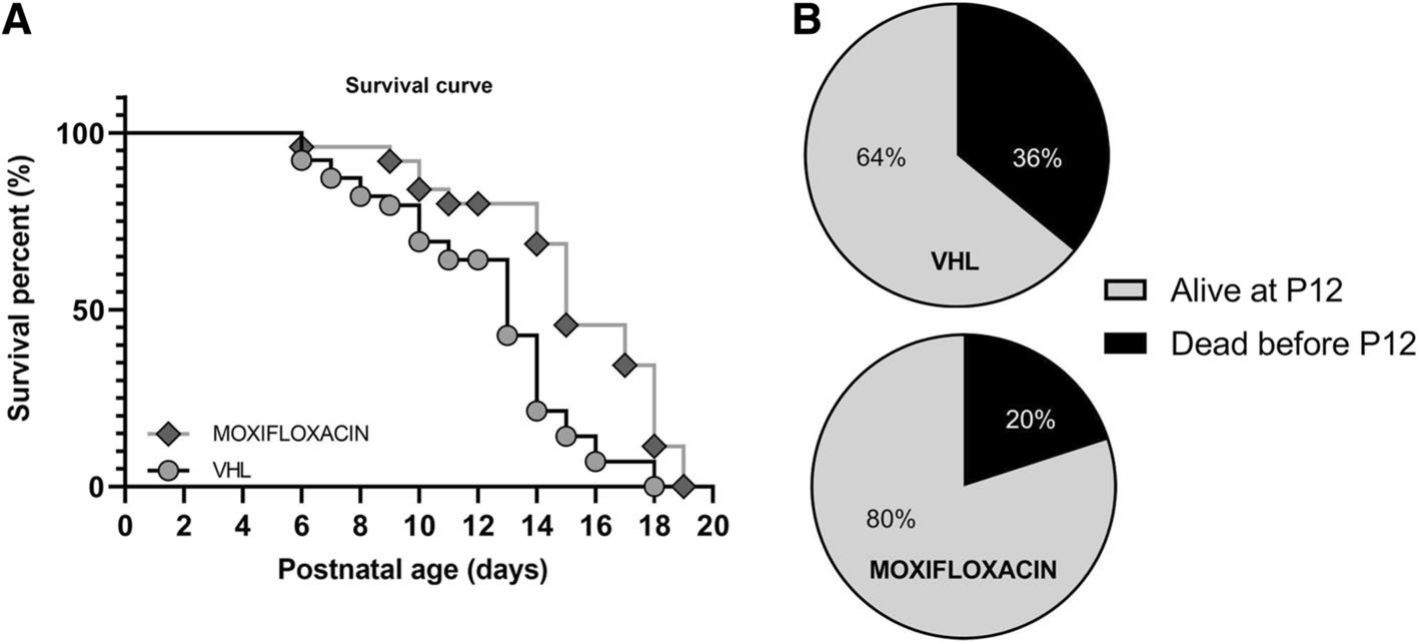
Effect of moxifloxacin on the delta7 mice survival. **A** Survival analysis in treated delta 7 mice. Kaplan–Meier survival curve shows that moxifloxacin can significantly extend delta 7 mice lifespan (VHL *n* = 39, moxifloxacin *n* = 25). Statistical analysis: Kaplan–Meier curve test (censoring the mice that were sacrificed at P12), Log-rank Mantel Cox post hoc test, *p* < 0.05. **B** Percentages of VHL and moxifloxacin-treated delta 7 mice that were alive at P12 or prematurely died (VHL *n* = 39, moxifloxacin *n* = 25)

Finally, we assessed whether the treatment could also extend the lifespan of the treated mice. Indeed, we observed that the administration of moxifloxacin significantly increased the lifespan of treated mice (Kaplan–Meier curve test, Log-rank Mantel–Cox post hoc test, *p < 0.05), whose median survival was 15.4% higher compared to vehicle-treated (VHL) group (Fig. 7A; median survival SMA moxifloxacin = 15 days, SMA VHL = 13 days). To further corroborate such result, we also evaluated the percentage of SMA mice that reached the age of P12, compared to those that spontaneously died earlier: only 20% of moxifloxacin-treated pups died before P12 vs 36% of VHL mice (Fig. 7B). Interestingly, the moxifloxacin mice always exhibited greater motor strength and activity than VHL (Supplementary Video 1). This further underlines the drug effect in ameliorating the survival and wellbeing of treated mice.

## Discussion

We have previously shown that moxifloxacin increases the SMN protein levels in SMA fibroblasts by boosting *SMN2* exon 7 inclusion. We also observed an increase of SRSF1 expression, the main exon 7 splicing enhancer, and related that to the moxifloxacin-triggered inhibition of topoisomerase II (TOP2) [15]. There is a precedence for fluoroquinolones influencing splicing by such mechanism of action, such as in the case of caspase 2 splicing, where the increased exon 9 inclusion is most likely caused by increased expression of regulatory splicing factors as a consequence of TOP2 inhibition [31, 32]. It is possible that the moxifloxacin triggered inhibition of the TOP2A results in a quick and global release of polymerase II from promoter–proximal pausing, which results in the upregulation of interdependent transcription and splicing factor [31, 32].

As a continuation of our previous work in fibroblasts, we show that moxifloxacin also increased the *SMN2* exon 7 inclusion in SMA type I and type II MNs. That was consequent with increased SMN levels and almost complete rescue of the SMA MNs degeneration. Interestingly, the treatment restored only around 30% of SMN normal levels in a healthy control cell line, but the rescue of MN degeneration was around 90% at some concentrations. This indicated that even a relatively discreet increase in SMN protein can significantly impact the phenotypes of the cell model, and moxifloxacin treatment did overcome such threshold. We have observed that higher concentrations still significantly increases the SMN protein levels but do not protect the MNs from degeneration as it would be expected, indicating a potential toxic effect of the drug. Notably, the survival rate and the SMN protein levels after moxifloxacin treatment are above the results obtained with the current first-line drug risdiplam.

These promising in vitro results were further investigated in vivo. To this aim, we evaluated the effects of the chronic administration of moxifloxacin in the most used and characterized type II SMA model, the delta 7 mice that reproduces the main disease hallmarks at both behavioural and cellular levels [26].

We confirmed that moxifloxacin was able to significantly increase SMN protein levels (both in the spinal cord and skeletal muscles), and consequently delay neurodegeneration, and improve muscle trophism and NMJ phenotypes in the SMA mice. Indeed, the SMN-dependent mechanism of action of moxifloxacin has been demonstrated by molecular and stereological analyses in the spinal cord of treated mice, where an increase in FL-SMN protein levels and the density of lower MNs in the lumbar spinal tract were observed, in comparison with controls. Moreover, according to previous evidence highlighting that the SMA pathophysiology is correlated with altered levels of apoptosis-related proteins (e.g., Bax, Bcl-xL, Bcl2) [12, 27, 33–36], we observed a significant reduction of cells positive to Cleaved Caspase 3 (a pro-apoptotic marker) in moxifloxacin-treated mice compared to VHL pups, confirming the efficacy of the drug in modulating cell death mechanisms.

We also observed a significant decrease in the astrocyte activation at the spinal cord level. On one side, this could depend on the reduced neurodegeneration observed since these two processes, cell death and neuroinflammation, are strongly intertwined [37–39]. On the other side, interestingly, it has been reported that the restoration of SMN in MNs can only determine slightly enhanced recovery and survival of SMA mice, while a winning strategy could be increasing SMN protein levels also in other cellular populations, such as astrocytes, which dysfunction is known to exacerbate SMA condition [28, 40, 41]. Moreover, in addition to the splicing modifier role of moxifloxacin, the fluoroquinolone family can exert immunomodulatory and anti-inflammatory activities per se, by inhibiting the production of a number of pro-inflammatory cytokines (as IL‑1, TNF‑α, IL-6 and IL-8) [42–45] and reducing neuroinflammation at the CNS level [46].

Concerning the skeletal muscles, we first analysed the SMN expression, whose presence is pivotal for their correct functioning. Indeed, in 2020, Kim and coll. demonstrated that the selective SMN deprivation in these tissues can determine muscle fiber defects, NMJ abnormalities, and compromised motor performance [3]. We observed that moxifloxacin treatment significantly increased SMN protein levels in the quadriceps, while only a positive trend was visible in the gastrocnemius compared to controls. Accordingly, the analysed morphological parameters (i.e., fiber size, perimeter and Feret's diameters [30] indicated a significantly enhanced trophism only for the quadriceps slices. These differences could depend on the type of muscle. Indeed, in the case of SMA, proximal muscles (as quadriceps) are earlier affected and to a greater extent than the distal ones (as gastrocnemius) due to the selective vulnerability of MNs innervating proximal muscles [47]. This could suggest that moxifloxacin effects on trophism are more evident in the most affected muscles, while in the others (“more resistant”), it is possible to see only a positive trend. These observations were also partially confirmed by the NMJ analyses performed, which again highlighted the most remarkable improvements in the quadriceps compared to the gastrocnemius. Indeed, in the latter one, the percentage of denervated NMJs was unchanged compared to the VHL group, whereas significantly reduced in the quadriceps. The dissimilarity between the two muscles could also stem from different expression levels of moxifloxacin´s primary target, i.e., TOP2A. The RNA-seq data available (http://muscledb.org/) shows that the TOP2A expression in gastrocnemius is twice that in quadriceps. This means that higher moxifloxacin concentration is expected to be needed in gastrocnemius to effectively repress TOP2. We also analyzed the diaphragm of the moxifloxacin-treated mice, and we have not detected any change in the SMN levels (Supplementary Fig. 4). This is consistent with our hypothesis since the TOP2A levels in the diaphragm are triple compared to the quadriceps. Initially, the levels of main *SMN2* splicing factors in gastrocnemius and quadriceps are similar, according to the database.

In contrast, by analysing the innervation/maturation of endplates, we noticed that both muscles showed an improvement, characterized by a significantly higher percentage of mono-innervated NMJs. As known, in SMA mouse models, the formation of the NMJs proceeds normally, but their maturation stalls [48], and the lack of SMN seems to have a central role in both processes [reviewed in [25]]. Therefore, we can speculate that by enhancing the SMN production and improving the reciprocal signalling between MNs and muscles [in turn important for the formation and maintenance of NMJs [49]], moxifloxacin successfully contributed to sustaining the endplate maturation progress.

Overall, such muscle and NMJ phenotype ameliorations justify the positive results obtained in the body weight assessment and the behavioural tests, highlighting the reduced muscle atrophy and the improved motor performances (in terms of posture, strength, and coordination), compared to the control group. We also performed a survival study, and although a certain variability in the disease progression has been reported for this mouse model [50], we observed a significant lifespan extension in treated mice compared to controls (+ 15%). Indeed, moxifloxacin-treated mice achieved a median lifespan of 15 days: this increase can be considered important related to the extremely short lifespan of delta 7 mice (on average 13 days for the VHL-treated group, in this work).

To our knowledge, compared to previously repositioned drugs with SMN-dependent effect (especially those influencing the alternative splicing of SMN2 pre-mRNA) [12, 51], the antibiotic moxifloxacin for the treatment of delta 7 mice resulted in higher reaching improvements in SMA condition rescuing, ranging from positive outcomes concerning survival and motor behaviour, SMN protein level increase in different tissues, to delayed neurodegeneration, modulated neuroinflammation and improved NMJ maturation/innervation. Preclinical data from patient-derived MNs and a severe SMA mouse model encourage the clinical repurposing of the molecule. Specifically, the degree of the rescue of MN survival is higher than risdiplam at the highest non-toxic concentration. However, we could not compare side by side risdiplam and moxifloxacin in similar experiments in vivo.

By validating moxifloxacin as a candidate drug for SMA, we prove that drug repositioning is a valuable strategy for discovering new therapies for rare diseases. In particular, repurposed drugs can reach patients faster than new molecules [12], and may target disease pathways from different perspectives, thus enriching therapeutic options either as stand-alone drugs or to complement therapies that have already reached human use authorization.

### Supplementary Information

Below is the link to the electronic supplementary material.

Supplementary file1 (AVI 4197 kb)

Supplementary file2 (DOCX 47 kb)

Supplementary file3 (DOCX 158 kb)

Supplementary file4 (DOCX 221 kb)

Supplementary file5 (DOCX 39 kb)

Supplementary file6 (DOCX 92 kb)

Supplementary file7 (DOCX 215 kb)

Supplementary file8 (DOCX 13260 kb)

Supplementary file9 (DOCX 13 kb)

Supplementary file10 (DOCX 13 kb)

## Data Availability

The data sets generated during and/or analysed during the current study are available from the corresponding author on reasonable request.

## References

[1] Wirth B, Karakaya M, Kye MJ, Mendoza-Ferreira N (2020). Twenty-five years of spinal muscular atrophy research: from phenotype to genotype to therapy, and what comes next. Annu Rev Genom Hum Genet.

[2] Shababi M, Lorson CL, Rudnik-Schoneborn SS (2014). Spinal muscular atrophy: a motor neuron disorder or a multi-organ disease?. J Anat.

[3] Kim J-K, Jha NN, Feng Z (2020). Muscle-specific SMN reduction reveals motor neuron–independent disease in spinal muscular atrophy models. J Clin Investig.

[4] Martinez-Hernandez R, Bernal S, Alias L, Tizzano EF (2014). Abnormalities in early markers of muscle involvement support a delay in myogenesis in spinal muscular atrophy. J Neuropathol Exp Neurol.

[5] Lefebvre S, Burglen L, Reboullet S (1995). Identification and characterization of a spinal muscular atrophy-determining gene. Cell.

[6] Lorson CL, Hahnen E, Androphy EJ, Wirth B (1999). A single nucleotide in the SMN gene regulates splicing and is responsible for spinal muscular atrophy. Proc Natl Acad Sci USA.

[7] Wadman RI, Stam M, Gijzen M (2017). Association of motor milestones, SMN2 copy and outcome in spinal muscular atrophy types 0–4. J Neurol Neurosurg Psychiatry.

[8] Mercuri E, Pera MC, Scoto M (2020). Spinal muscular atrophy: insights and challenges in the treatment era. Nat Rev Neurol.

[9] Vandamme C, Adjali O, Mingozzi F (2017). Unraveling the complex story of immune responses to AAV vectors trial after trial. Hum Gene Ther.

[10] Labianca L, Weinstein SL (2019). Scoliosis and spinal muscular atrophy in the new world of medical therapy: providing lumbar access for intrathecal treatment in patients previously treated or undergoing spinal instrumentation and fusion. J Pediatr Orthop B.

[11] Landfeldt E, Pechmann A, McMillan HJ (2021). Costs of illness of spinal muscular atrophy: a systematic review. Appl Health Econ Health Policy.

[12] Menduti G, Rasà DM, Stanga S, Boido M (2020). Drug screening and drug repositioning as promising therapeutic approaches for spinal muscular atrophy treatment. Front Pharmacol.

[13] Lukas T, Siddique T (2019). Cancer drug repurposing for treating amyotrophic lateral sclerosis (ALS) (S5.004). Neurology.

[14] Hoolachan JM, Sutton ER, Bowerman M (2019). Teaching an old drug new tricks: repositioning strategies for spinal muscular atrophy. Future Neurol.

[15] Konieczny P, Artero R (2020). Drosophila SMN2 minigene reporter model identifies moxifloxacin as a candidate therapy for SMA. FASEB J.

[16] Maury Y, Côme J, Piskorowski RA (2015). Combinatorial analysis of developmental cues efficiently converts human pluripotent stem cells into multiple neuronal subtypes. Nat Biotechnol.

[17] Mérien A, Tahraoui-Bories J, Cailleret M (2021). CRISPR gene editing in pluripotent stem cells reveals the function of MBNL proteins during human *in vitro* myogenesis. Hum Mol Genet.

[18] D’Amico D, Biondi O, Januel C (2022). Activating ATF6 in spinal muscular atrophy promotes SMN expression and motor neuron survival through the IRE1α-XBP1 pathway. Neuropathol Appl Neurobiol.

[19] Chaouch S, Mouly V, Goyenvalle A (2009). Immortalized skin fibroblasts expressing conditional MyoD as a renewable and reliable source of converted human muscle cells to assess therapeutic strategies for muscular dystrophies: validation of an exon-skipping approach to restore dystrophin in Duchenne muscular dystrophy cells. Hum Gene Ther.

[20] Edom F, Mouly V, Barbet JP (1994). Clones of human satellite cells can express in vitro both fast and slow myosin heavy chains. Dev Biol.

[21] Young J, Margaron Y, Fernandes M (2018). MyoScreen, a high-throughput phenotypic screening platform enabling muscle drug discovery. SLAS Discov.

[22] Valsecchi V, Boido M, De Amicis E (2015). Expression of muscle-specific MiRNA 206 in the progression of disease in a murine SMA Model. PLoS ONE.

[23] Meeker ND, Hutchinson SA, Ho L, Trede NS (2007). Method for isolation of PCR-ready genomic DNA from zebrafish tissues. Biotechniques.

[24] El-Khodor BF, Edgar N, Chen A (2008). Identification of a battery of tests for drug candidate evaluation in the SMNΔ7 neonate model of spinal muscular atrophy. Exp Neurol.

[25] Boido M, Vercelli A (2016). Neuromuscular junctions as key contributors and therapeutic targets in spinal muscular atrophy. Front Neuroanat.

[26] Le TT, Pham LT, Butchbach MER (2005). SMNΔ7, the major product of the centromeric survival motor neuron (SMN2) gene, extends survival in mice with spinal muscular atrophy and associates with full-length SMN. Hum Mol Genet.

[27] Piras A, Schiaffino L, Boido M (2017). Inhibition of autophagy delays motoneuron degeneration and extends lifespan in a mouse model of spinal muscular atrophy. Cell Death Dis.

[28] Rindt H, Feng Z, Mazzasette C (2015). Astrocytes influence the severity of spinal muscular atrophy. Hum Mol Genet.

[29] Bolliger MF, Zurlinden A, Lüscher D (2010). Specific proteolytic cleavage of agrin regulates maturation of the neuromuscular junction. J Cell Sci.

[30] Boido M, De Amicis E, Valsecchi V (2018). Increasing agrin function antagonizes muscle atrophy and motor impairment in spinal muscular atrophy. Front Cell Neurosci.

[31] Solier S, Lansiaux A, Logette E (2004). Topoisomerase I and II inhibitors control caspase-2 pre-messenger RNA splicing in human cells. Mol Cancer Res.

[32] Fabian, (2010). Moxifloxacin enhances etoposide-induced cytotoxic, apoptotic and anti-topoisomerase II effects in a human colon carcinoma cell line. Int J Oncol.

[33] Tsai L-K, Tsai M-S, Ting C-H (2008). Restoring Bcl-xL levels benefits a mouse model of spinal muscular atrophy. Neurobiol Dis.

[34] Tsai MS, Chiu YT, Wang SH (2006). Abolishing bax-dependent apoptosis shows beneficial effects on spinal muscular atrophy model mice. Mol Ther.

[35] Garcera A, Mincheva S, Gou-Fabregas M (2011). A new model to study spinal muscular atrophy: neurite degeneration and cell death is counteracted by BCL-XL Overexpression in motoneurons. Neurobiol Dis.

[36] Schellino R, Boido M, Borsello T, Vercelli A (2018). Pharmacological c-Jun NH_2_-terminal kinase (JNK) pathway inhibition reduces severity of spinal muscular atrophy disease in mice. Front Mol Neurosci.

[37] Guadagno J, Xu X, Karajgikar M (2013). Microglia-derived TNFα induces apoptosis in neural precursor cells via transcriptional activation of the Bcl-2 family member Puma. Cell Death Dis.

[38] Heckmann BL, Tummers B, Green DR (2019). Crashing the computer: apoptosis vs. necroptosis in neuroinflammation. Cell Death Differ.

[39] Semmler A, Okulla T, Sastre M (2005). Systemic inflammation induces apoptosis with variable vulnerability of different brain regions. J Chem Neuroanat.

[40] Zhou C, Feng Z, Ko C-P (2016). Defects in motoneuron-astrocyte interactions in spinal muscular atrophy. J Neurosci.

[41] Abati E, Citterio G, Bresolin N (2020). Glial cells involvement in spinal muscular atrophy: Could SMA be a neuroinflammatory disease?. Neurobiol Dis.

[42] Barman Balfour JA, Lamb HM (2000). Moxifloxacin: a review of its clinical potential in the management of community-acquired respiratory tract infections. Drugs.

[43] Qiu Z, Yuan H, Li N (2018). Bidirectional effects of moxifloxacin on the pro-inflammatory response in lipopolysaccharide-stimulated mouse peritoneal macrophages. Mol Med Report.

[44] Radtke KK, Hesseling AC, Winckler JL (2021). Moxifloxacin pharmacokinetics, cardiac safety, and dosing for the treatment of rifampicin-resistant tuberculosis in children. Clin Infect Dis.

[45] Zusso M, Lunardi V, Franceschini D (2019). Ciprofloxacin and levofloxacin attenuate microglia inflammatory response via TLR4/NF-kB pathway. J Neuroinflamm.

[46] Kellermann K, Dertinger N, Blobner M (2011). Perioperative moxifloxacin treatment in rats subjected to deep hypothermic circulatory arrest: reduction in cerebral inflammation but without improvement in cognitive performance. J Thorac Cardiovasc Surg.

[47] d’Errico P, Boido M, Piras A (2013). Selective vulnerability of spinal and cortical motor neuron subpopulations in delta7 SMA mice. PLoS ONE.

[48] Kariya S, Park G-H, Maeno-Hikichi Y (2008). Reduced SMN protein impairs maturation of the neuromuscular junctions in mouse models of spinal muscular atrophy. Hum Mol Genet.

[49] Fralish Z, Lotz EM, Chavez T (2021). Neuromuscular development and disease: learning from in vitro and in vivo models. Front Cell Dev Biol.

[50] El-Khodor BF, Cirillo K, Beltran JA (2012). Prediction of death in the SMNΔ7 mouse model of spinal muscular atrophy: insight into disease stage and progression. J Neurosci Methods.

[51] Servais L, Baranello G, Scoto M (2021). Therapeutic interventions for spinal muscular atrophy: preclinical and early clinical development opportunities. Expert Opin Investig Drugs.

