# Supplementary material for: Moxifloxacin rescues SMA phenotypes in patient-derived cells and animal model
Source: Cell Mol Life Sci. 2022 Jul 22;79(8):441. doi: 10.1007/s00018-022-04450-8 (PMC9304069; doi:10.1007/s00018-022-04450-8)
Supplement: Supplementary file 2 — Supplementary file2 (DOCX 47 kb) [file 18_2022_4450_MOESM2_ESM.docx]

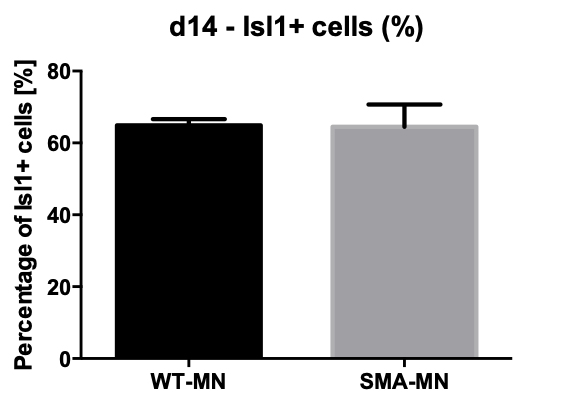


**Supplementary Figure 1. hiPSC-derived MNs from WT and SMA lines. Quantification of the Islet positive cells after 14 days of differentiation.**
