# Supplementary material for: Moxifloxacin rescues SMA phenotypes in patient-derived cells and animal model
Source: Cell Mol Life Sci. 2022 Jul 22;79(8):441. doi: 10.1007/s00018-022-04450-8 (PMC9304069; doi:10.1007/s00018-022-04450-8)
Supplement: Supplementary file 3 — Supplementary file3 (DOCX 158 kb) [file 18_2022_4450_MOESM3_ESM.docx]

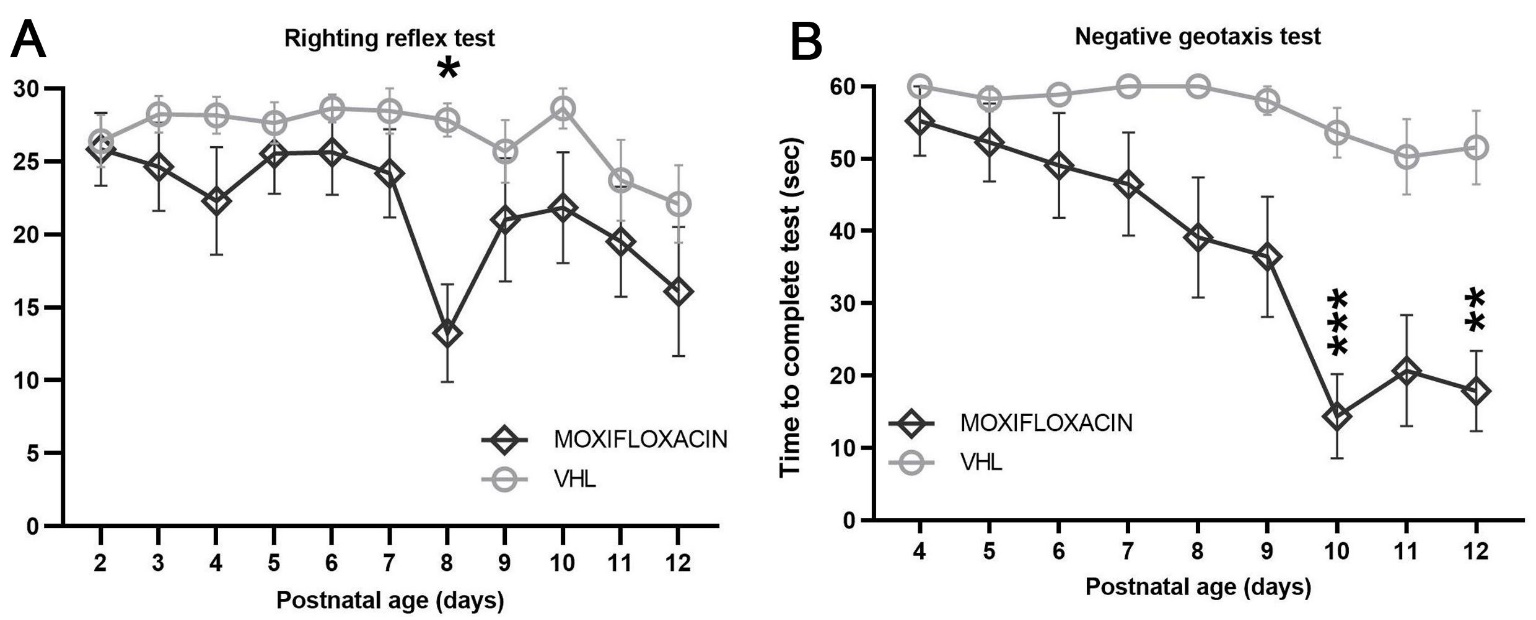


**Supplementary Figure 2. Moxifloxacin treatment shortens the time for delta 7 mice to complete behavioral motor tests.** (A) Righting reflex of treated delta 7 mice. Data are expressed as mean ± SEM, VHL n=21, moxifloxacin n=9, Statistical analysis: es model with Geisser-Greenhouse correction (F_(1,28)_ = 16.77) followed by Sidak’s multiple comparison post hoc test; * p<0.05. (B) Negative geotaxis of treated delta 7 mice. Data are expressed as mean ± SEM, VHL n=21, moxifloxacin n=9, Statistical analysis: mixed-effects model with Geisser-Greenhouse correction (F_(1, 28)_ = 46.13) followed by Sidak’s multiple comparison post hoc test; ** p<0.01, *** p<0.005.
