# Supplementary material for: Moxifloxacin rescues SMA phenotypes in patient-derived cells and animal model
Source: Cell Mol Life Sci. 2022 Jul 22;79(8):441. doi: 10.1007/s00018-022-04450-8 (PMC9304069; doi:10.1007/s00018-022-04450-8)
Supplement: Supplementary file 4 — Supplementary file4 (DOCX 221 kb) [file 18_2022_4450_MOESM4_ESM.docx]

**
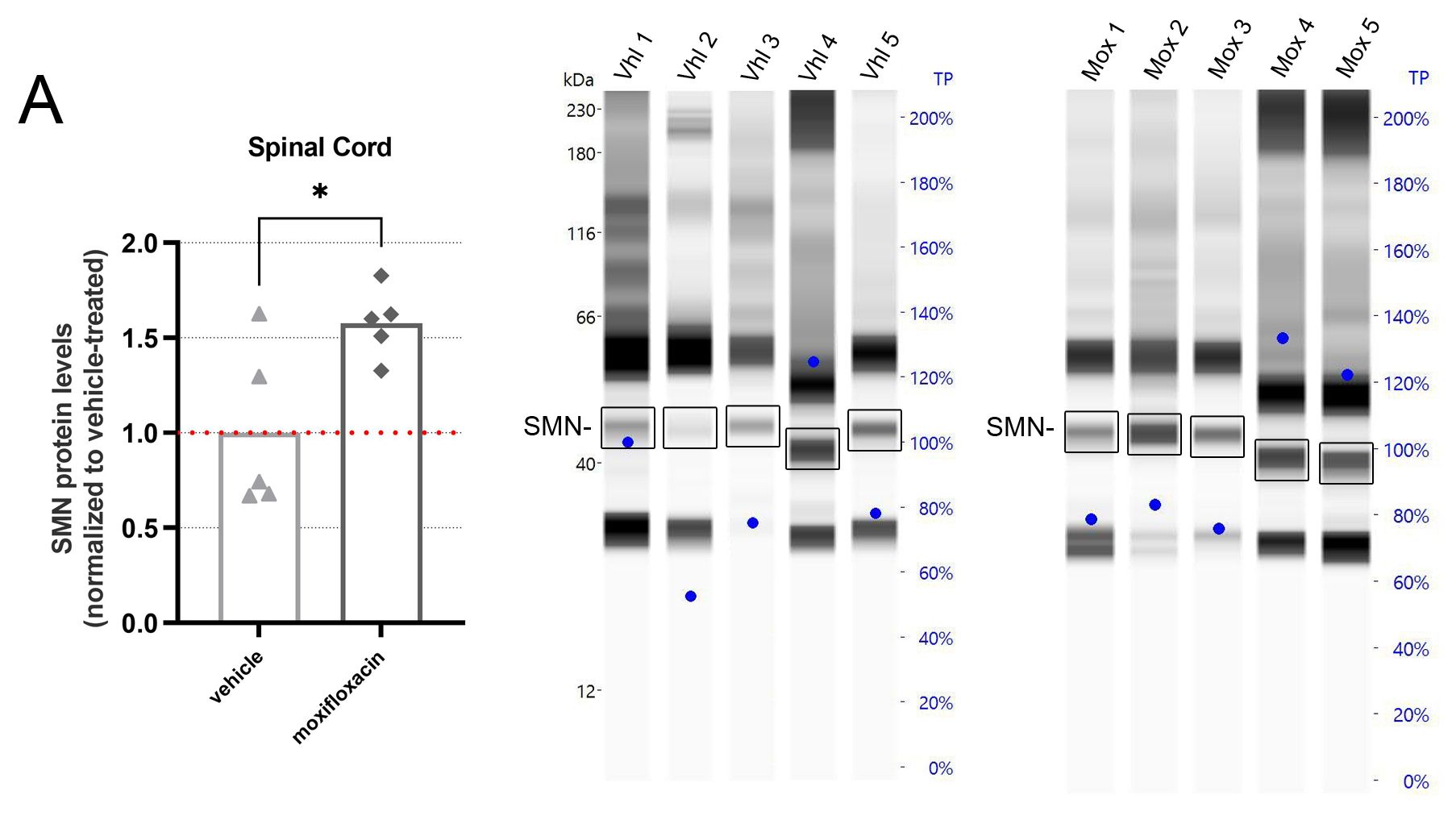

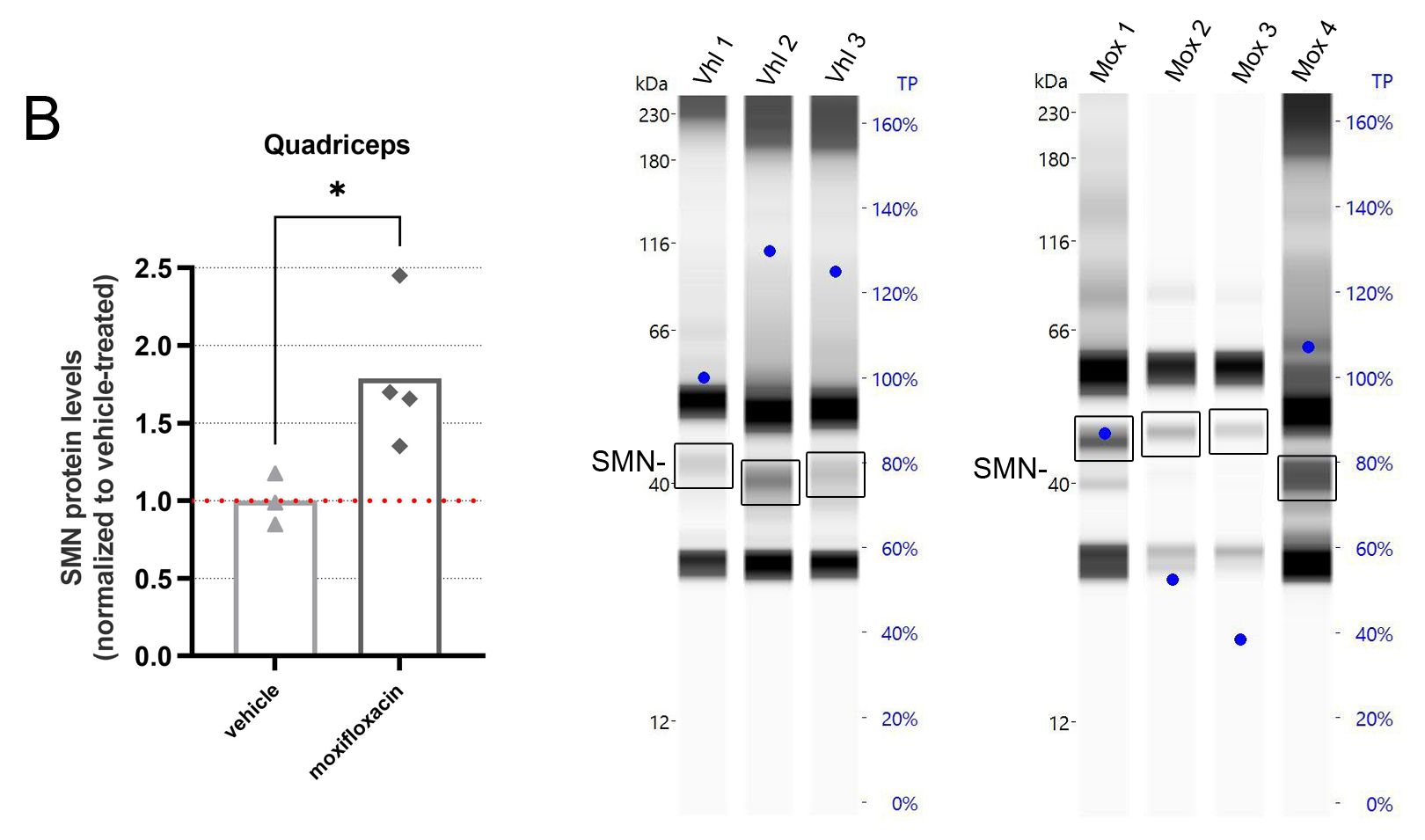
**

**Supplementary Figure 3. Capillary protein immunodetection analysis of SMN levels from Δ7 SMA mice treated with moxifloxacin.** SMN levels from spinal cord (A) and quadriceps (B) samples. The boxes in the lanes mark the SMN band used for analysis, while the blue dots indicate the total protein (TP) levels to which the SMN amount was normalized. The amount of the total protein of each sample is compared to the Vhl1 (100%). The graph shows the increase of SMN protein levels after moxifloxacin treatment compared to te vehicle-treated animals. The statistical analysis was performed usind Student´s t-test (*<p=0.05).
