# Supplementary material for: Moxifloxacin rescues SMA phenotypes in patient-derived cells and animal model
Source: Cell Mol Life Sci. 2022 Jul 22;79(8):441. doi: 10.1007/s00018-022-04450-8 (PMC9304069; doi:10.1007/s00018-022-04450-8)
Supplement: Supplementary file 5 — Supplementary file5 (DOCX 39 kb) [file 18_2022_4450_MOESM5_ESM.docx]

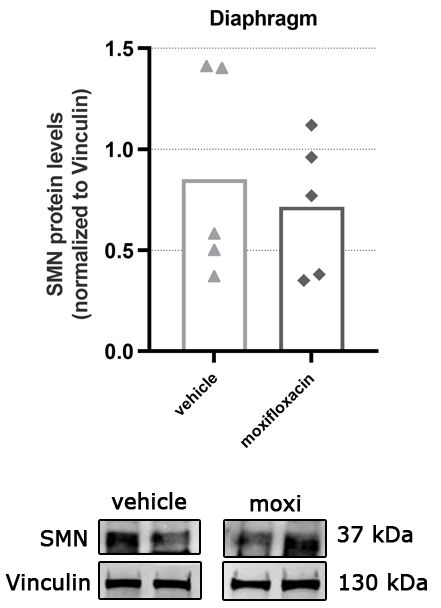


**Supplementary Figure 4. Representative immunoblots of SMN protein levels in VHL and moxifloxacin treated diaphragm.** The intensity of the bands of the SMN protein was calculated by referring to Vinculin protein levels (used as loading control). Data are expressed as mean ± SEM, n = 5 per group, Student’s t-test.
