# Supplementary material for: Moxifloxacin rescues SMA phenotypes in patient-derived cells and animal model
Source: Cell Mol Life Sci. 2022 Jul 22;79(8):441. doi: 10.1007/s00018-022-04450-8 (PMC9304069; doi:10.1007/s00018-022-04450-8)
Supplement: Supplementary file 6 — Supplementary file6 (DOCX 92 kb) [file 18_2022_4450_MOESM6_ESM.docx]

**Supplementary Figure MM1**. **Comparison of SMN expression level in hiPSC-derived MN from control SMA-type I and SMA-type II cell lines.** A) Relative expression level of SMN exon 7 and total SMN in control and SMA hiPSC-derived MN. B) Western blot analysis of SMN protein expression in control and SMA MNs. β-Actin was used as a housekeeping protein for normalization. C) HTRF analysis of the SMN protein level in control and SMA hiPSC-derived MNs. Data represent the mean values ± SD from 3 independent experiments in 2 controls (C03 in black and GM0314 in blue), 1 SMA-type I (grey) and 1 SMA-type II (red) cell lines. Statistics were calculated using an ordinary One-Way ANOVA, Turkey’s multiple comparisons test (p>0.05, ns: not significant, **p<0.01, ***p<0.001, ****p<0.0001).
