# Supplementary material for: Moxifloxacin rescues SMA phenotypes in patient-derived cells and animal model
Source: Cell Mol Life Sci. 2022 Jul 22;79(8):441. doi: 10.1007/s00018-022-04450-8 (PMC9304069; doi:10.1007/s00018-022-04450-8)
Supplement: Supplementary file 7 — Supplementary file7 (DOCX 215 kb) [file 18_2022_4450_MOESM7_ESM.docx]

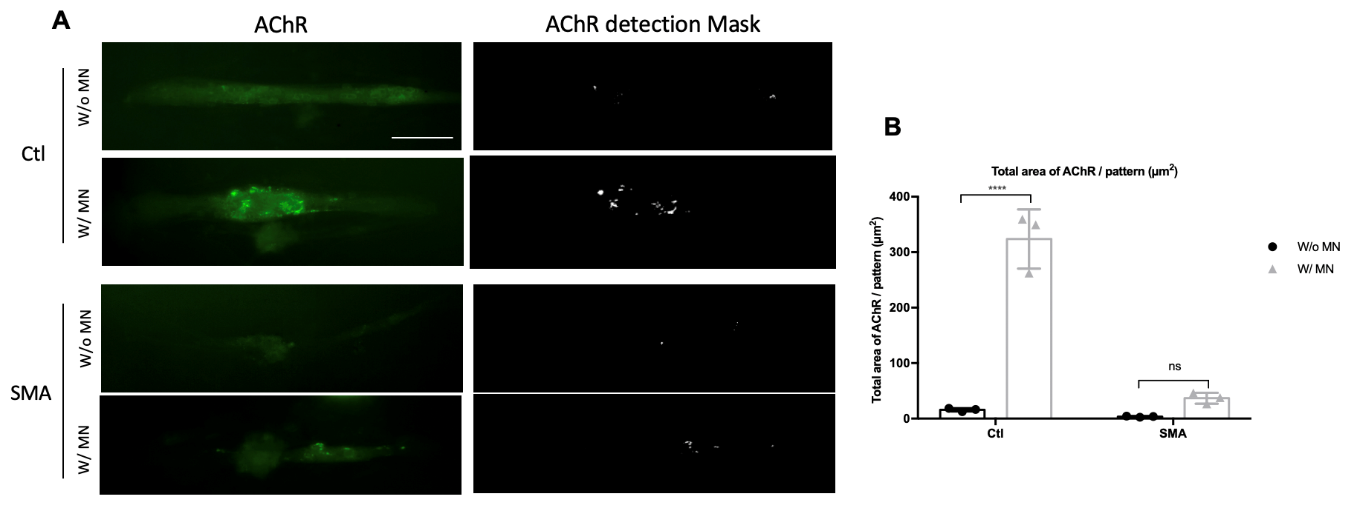


**Supplementary Figure MM2. hiPSC-derived MN enhanced AChR clustering after 7 days of coculture.** A) Representative examples of filter masks generated by an algorithm on Fiji Software for AChR detection based on representative images of AChR immunolabeling (in green). Scale bar 100 µm. B) Quantification of the total area of AChR per pattern is indicated on the histogram. Data represent the mean values ± SD from 3 experiments in control and SMA-type I co-culture. Statistics were calculated using an ordinary One-Way ANOVA, Tukey’s multiple comparisons test (p>0.05, ns: not significant, ****p<0.0001).
