# Supplementary material for: Moxifloxacin rescues SMA phenotypes in patient-derived cells and animal model
Source: Cell Mol Life Sci. 2022 Jul 22;79(8):441. doi: 10.1007/s00018-022-04450-8 (PMC9304069; doi:10.1007/s00018-022-04450-8)
Supplement: Supplementary file 8 — Supplementary file8 (DOCX 13260 kb) [file 18_2022_4450_MOESM8_ESM.docx]

**Supplementary Figure MM3. Uncropped western blot membranes.** Red box marks the signal used for the quantification of the SMN protein or the Vinculin control, blue box marks the samples obtained from the wild-type animals used as a positive control.

**
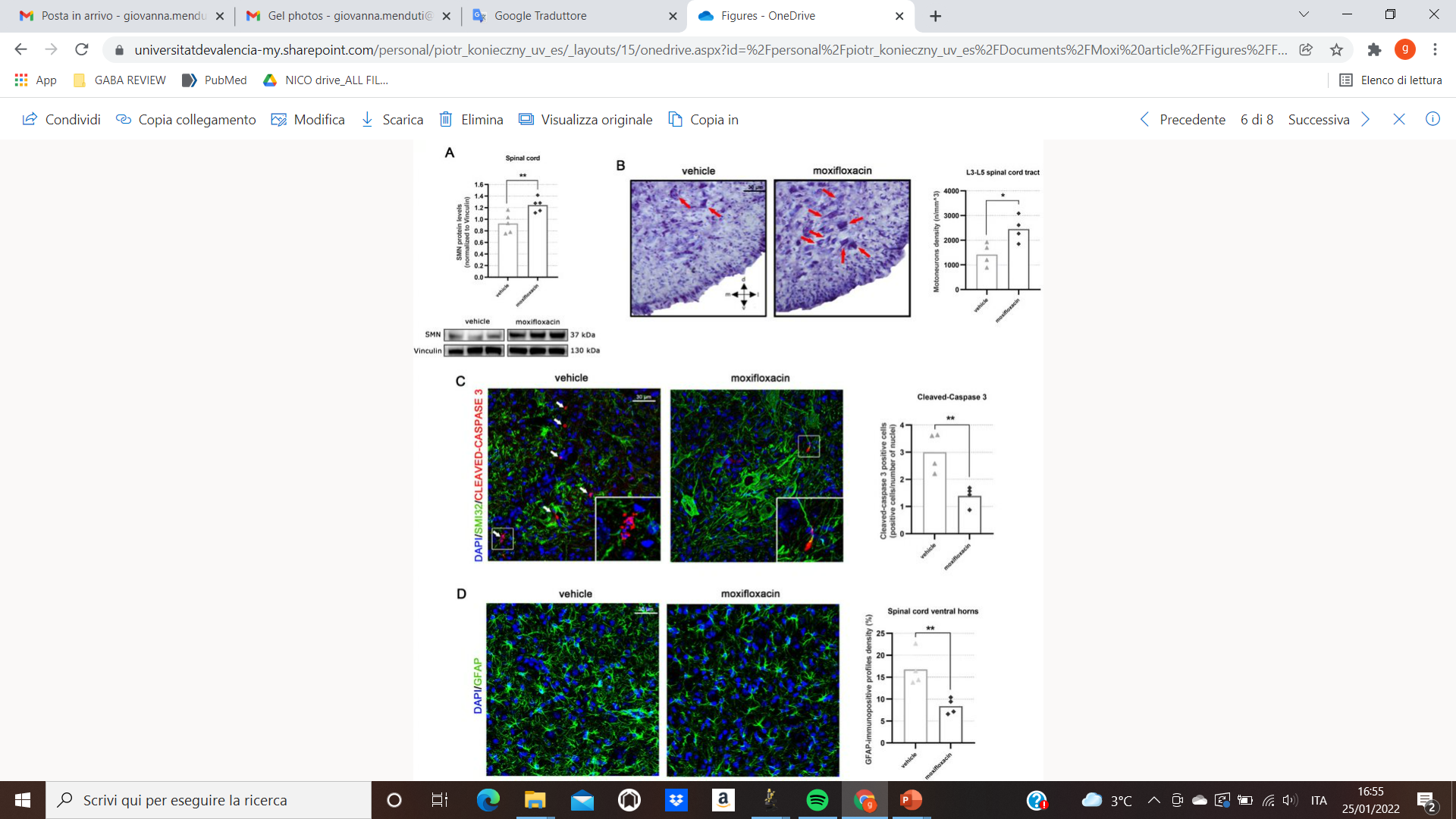
Figure 4A**


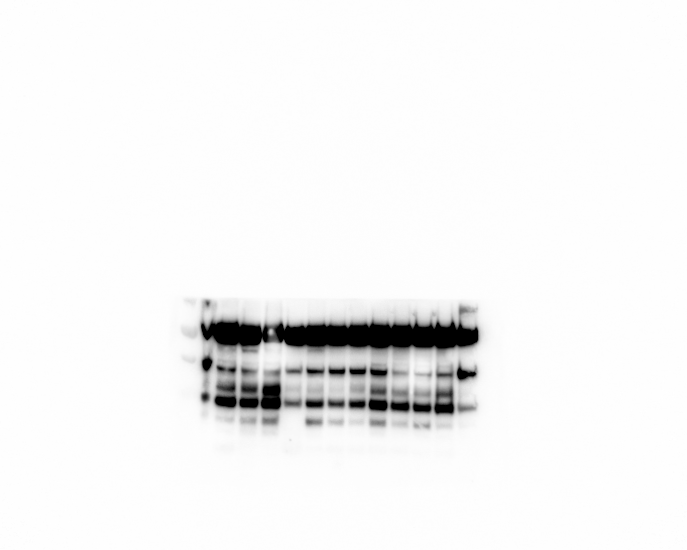

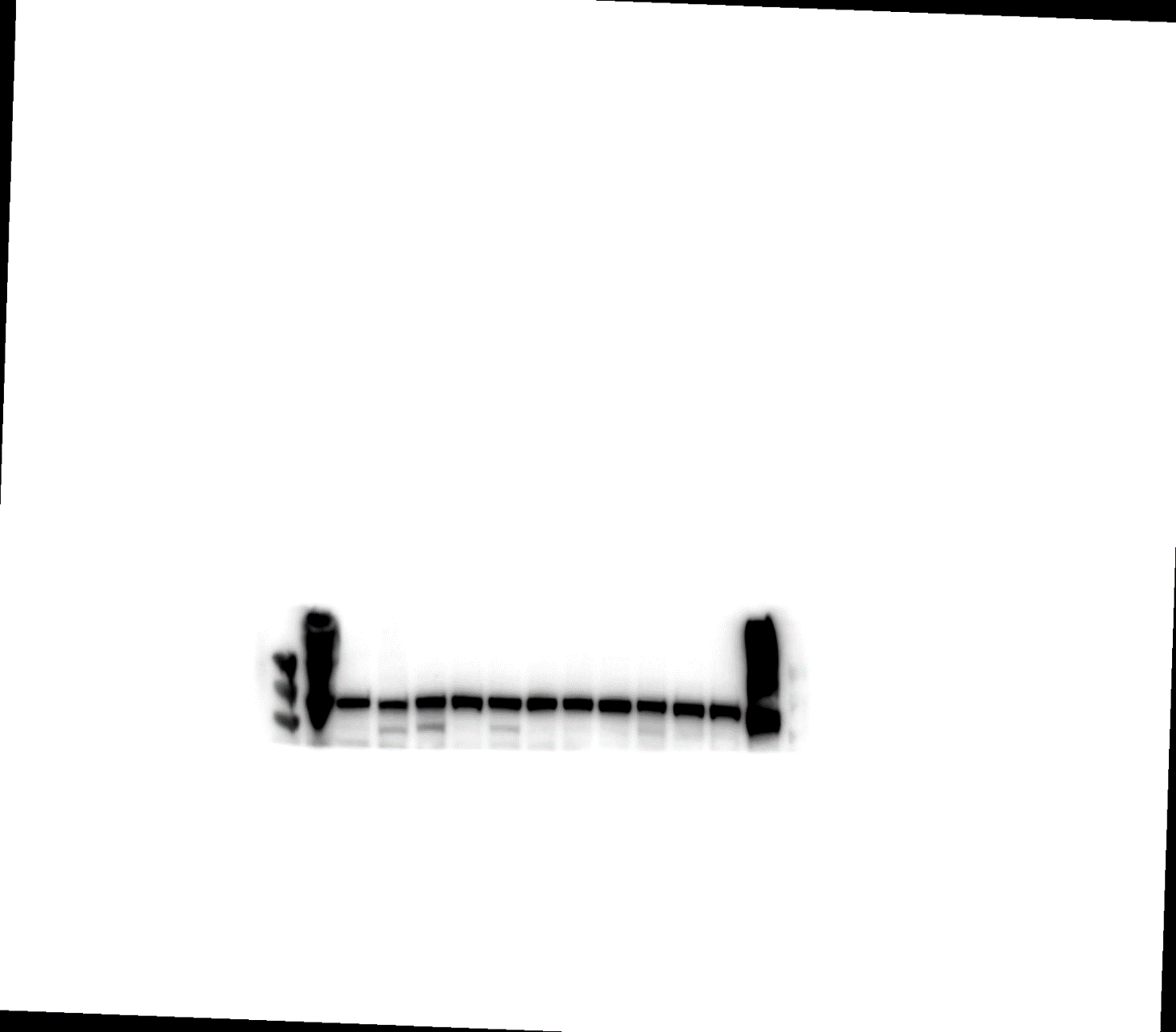


**SMN**

**VINCULIN**


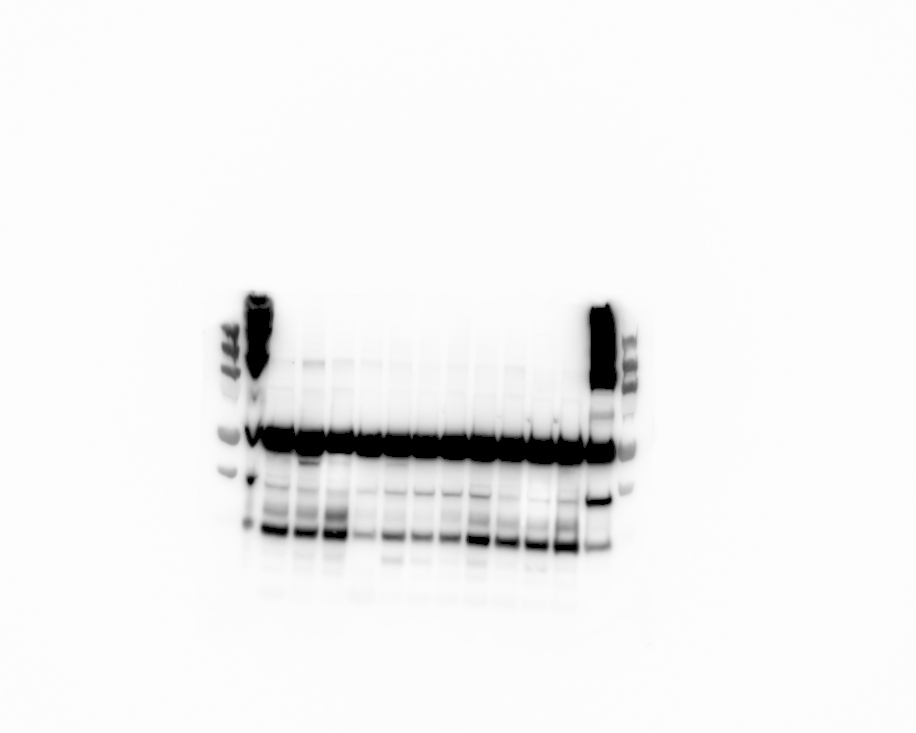

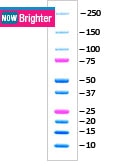

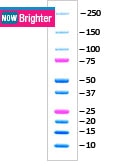

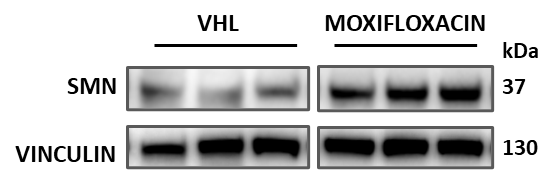

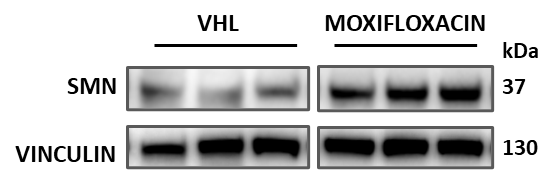

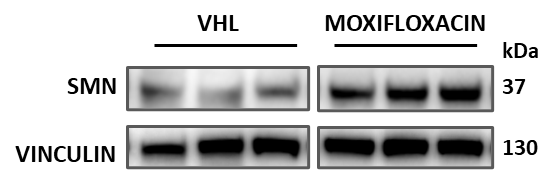

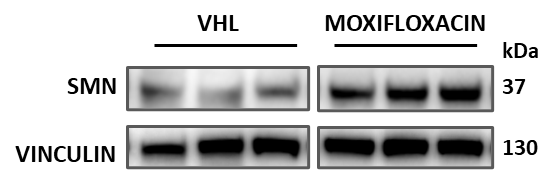


**
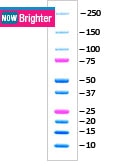
**

**Precision Plus
Protein Dual Color Standards**

**#1610374**

**
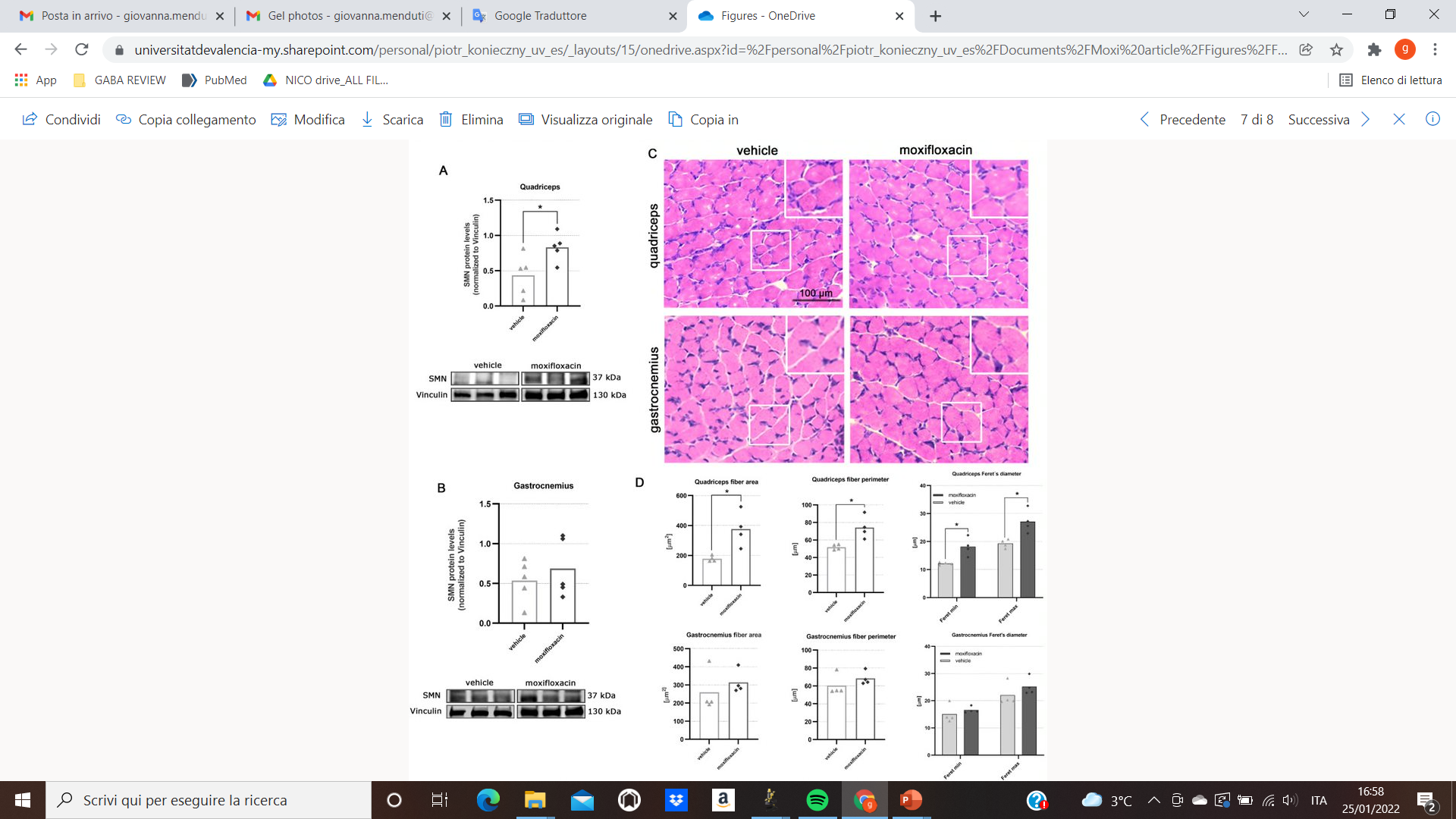
Figure 5A**


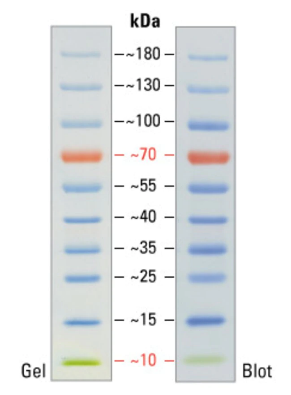

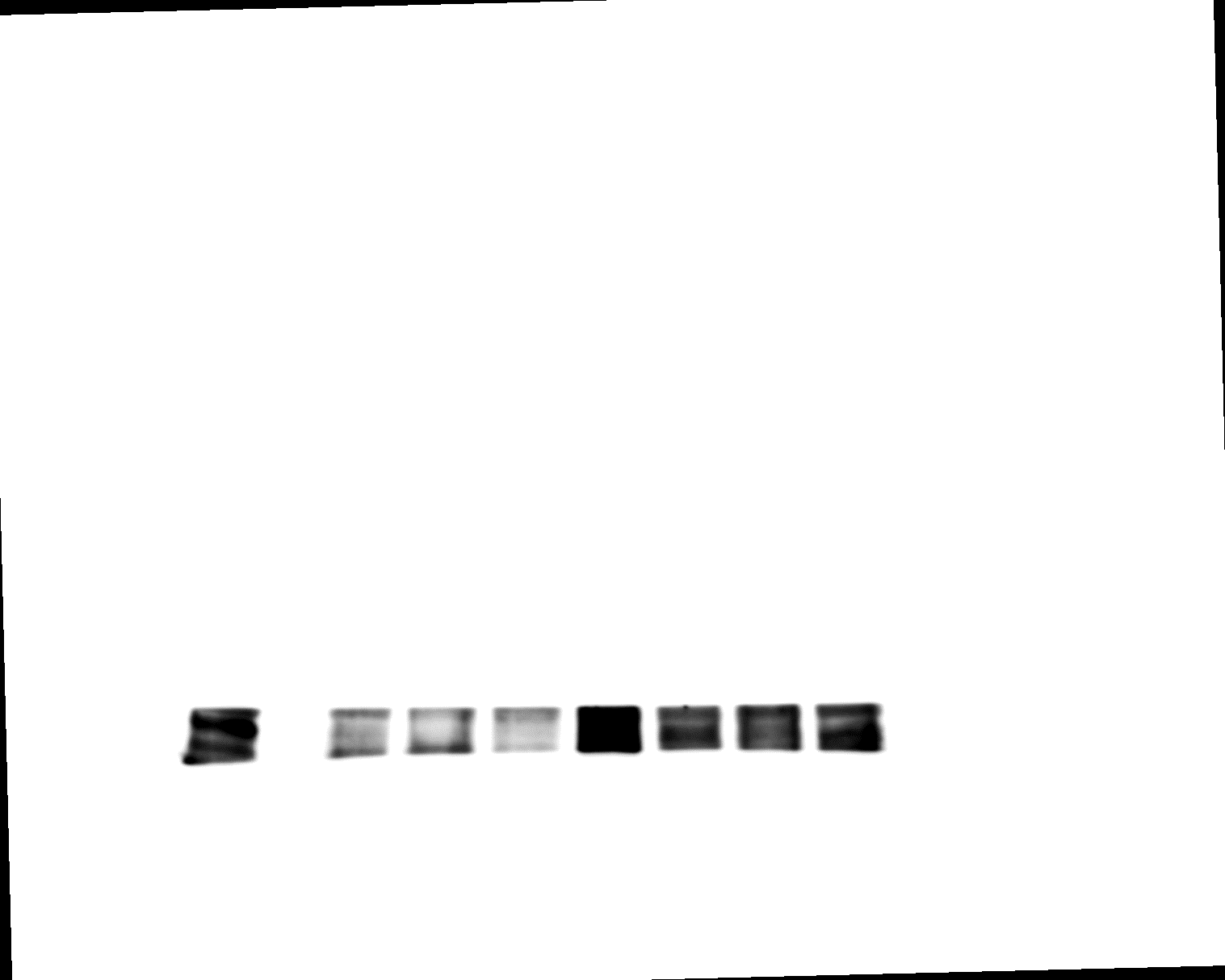

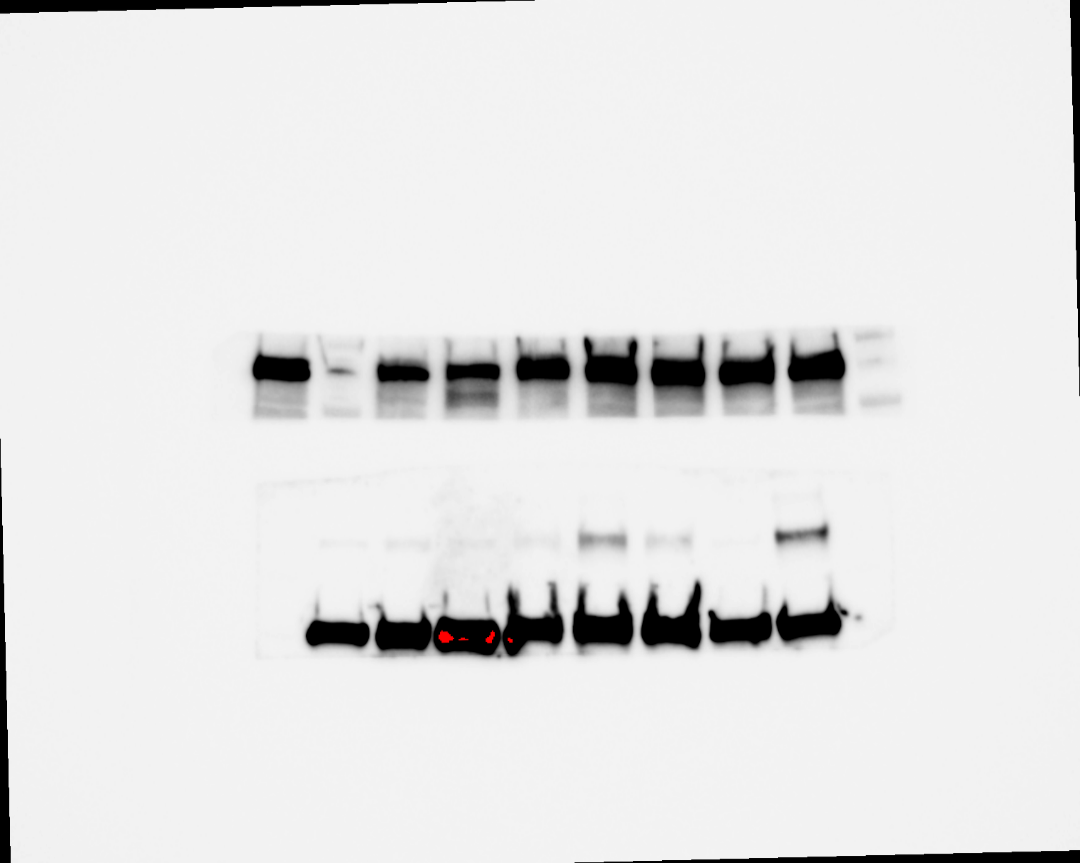


**SMN**

**VINCULIN**


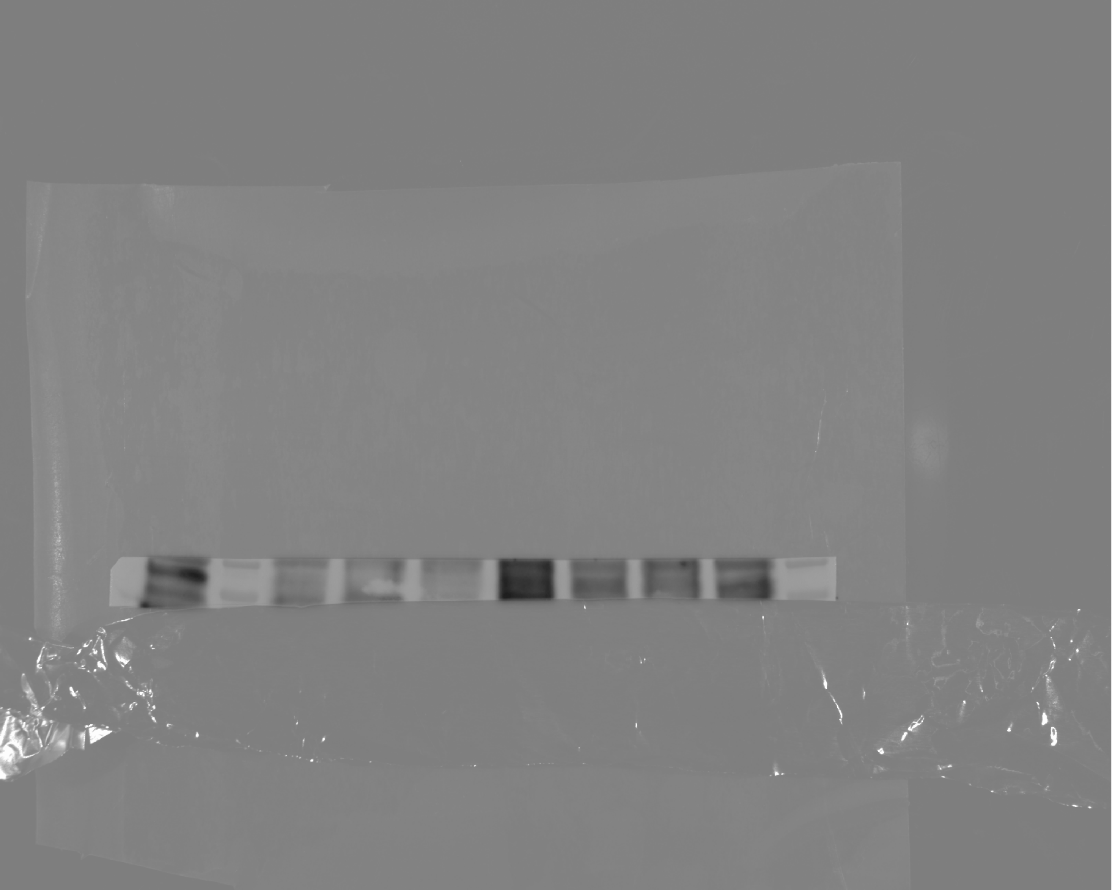


**37 kDa**

**130 kDa**


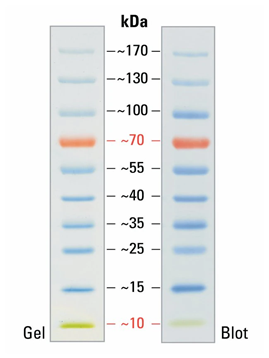

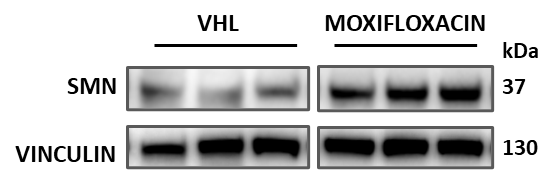

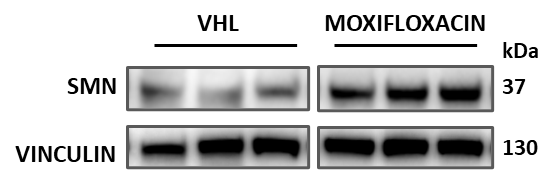

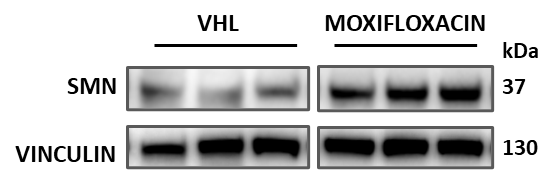

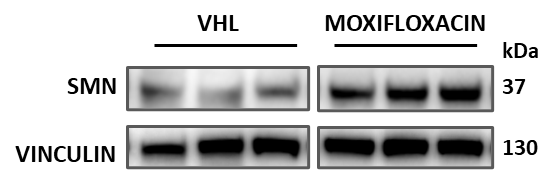


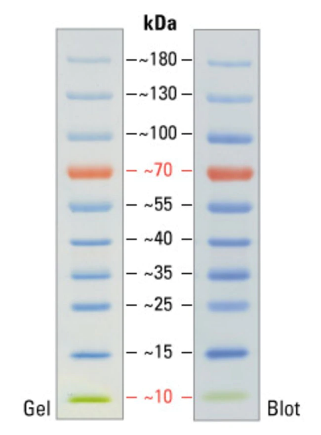


**pageruler prestained
protein ladder 26616**

**
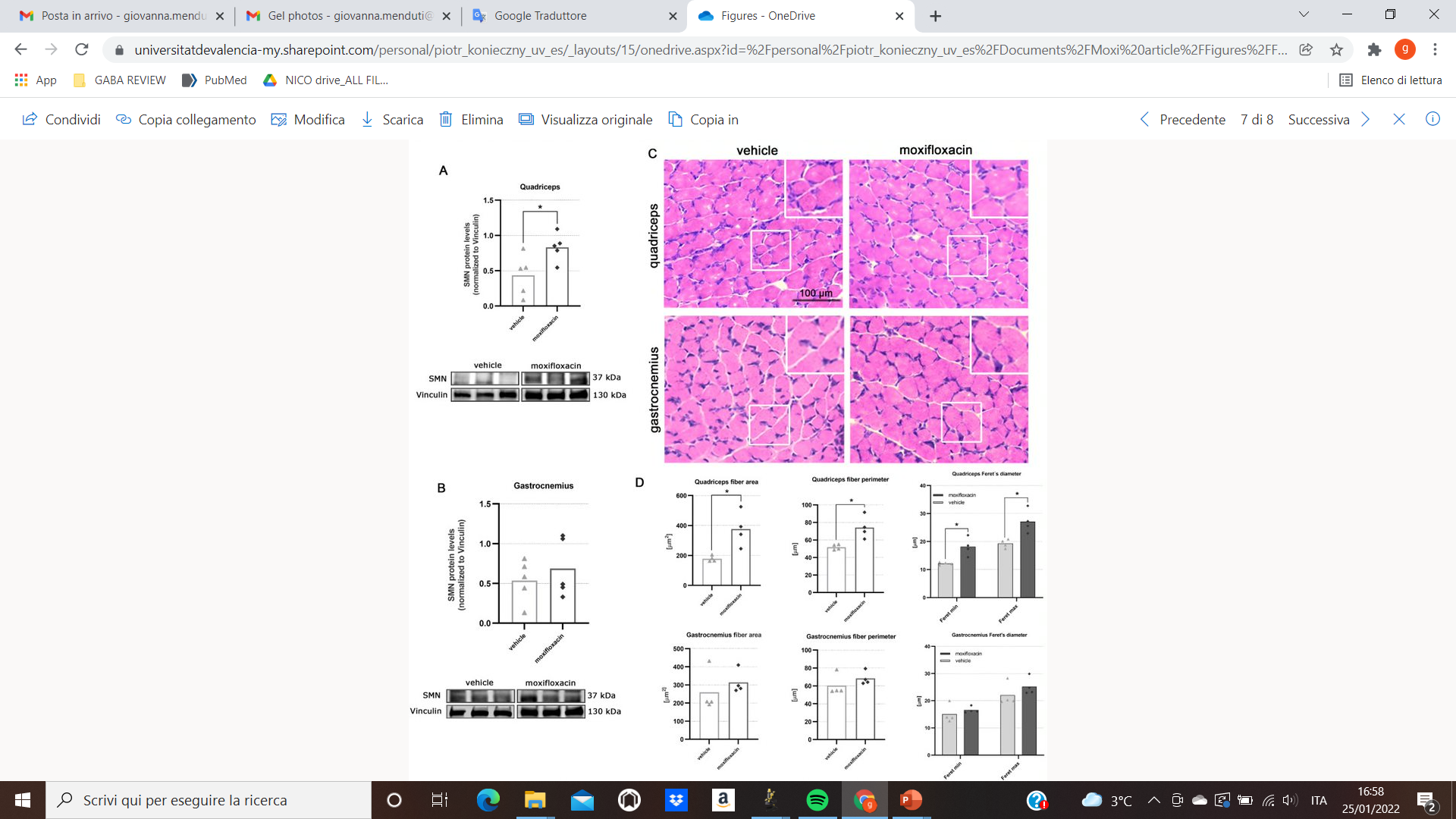
Figure 5B**


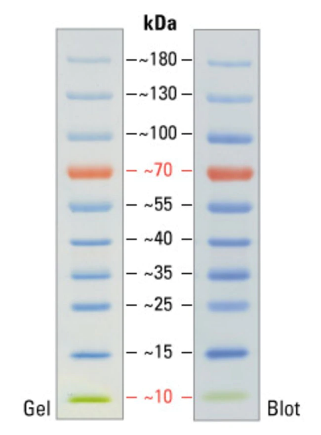


**130 kDa**


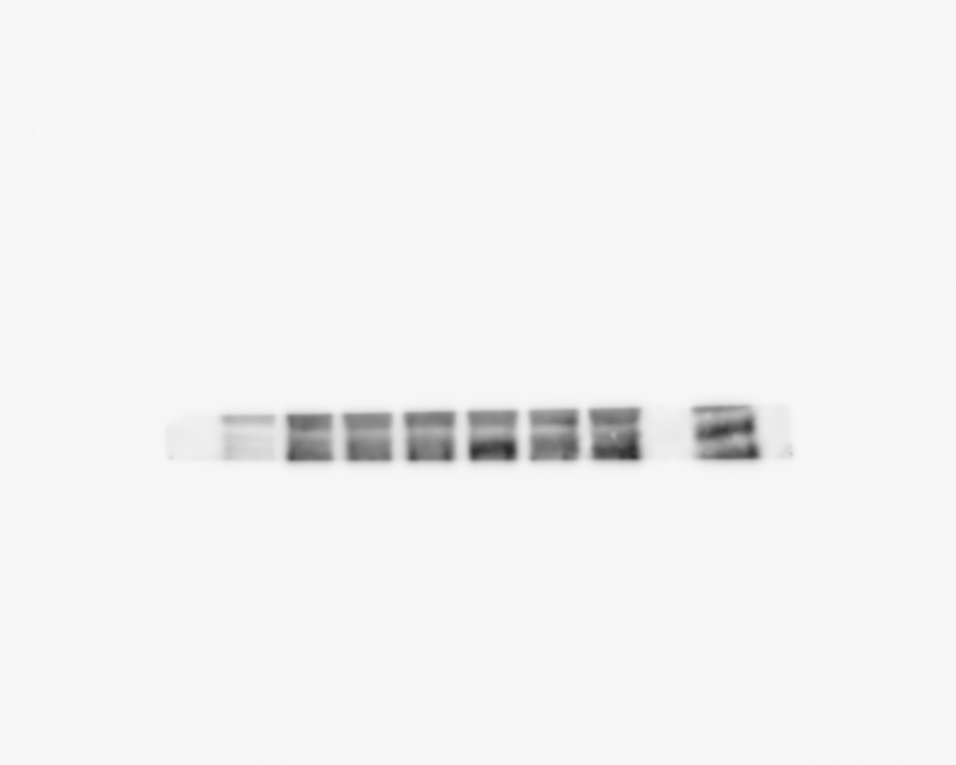

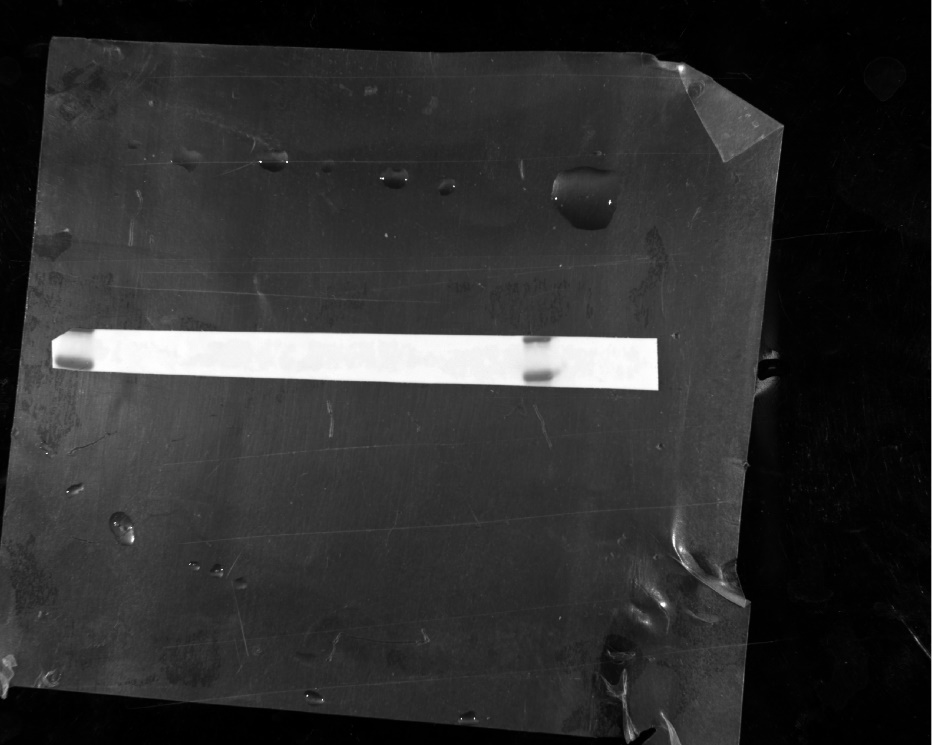


**SMN**

**VINCULIN**


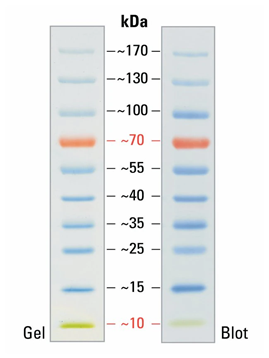


**37 kDa**


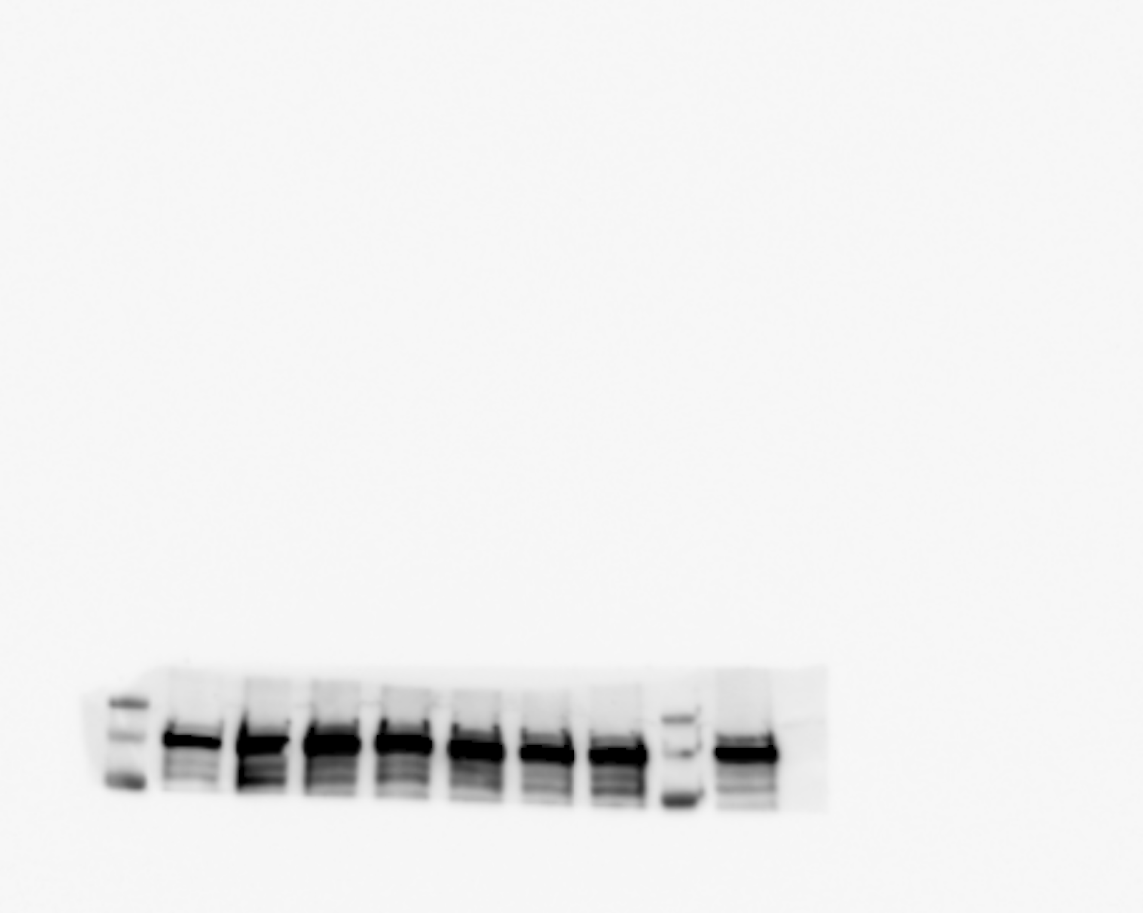

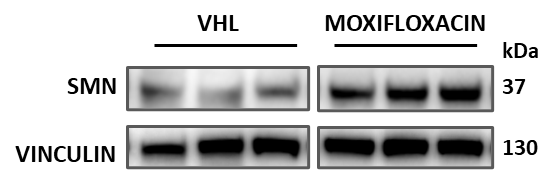

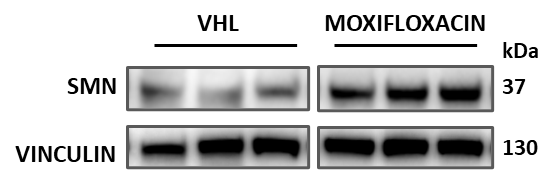

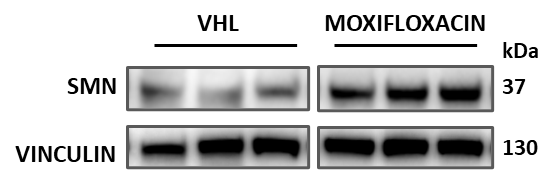

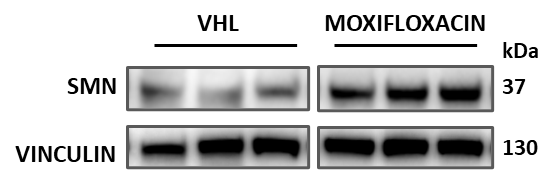


**
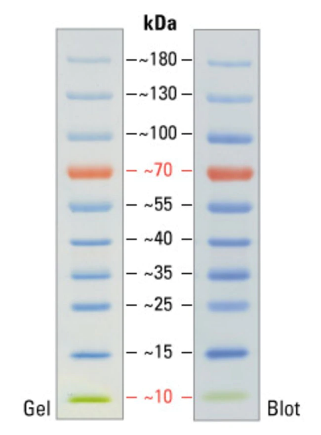
**

**pageruler prestained
protein ladder 26616**

**Supplementary Figure 4**

**
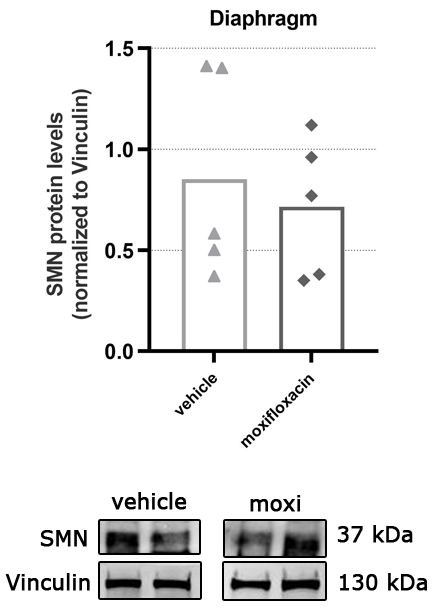
**


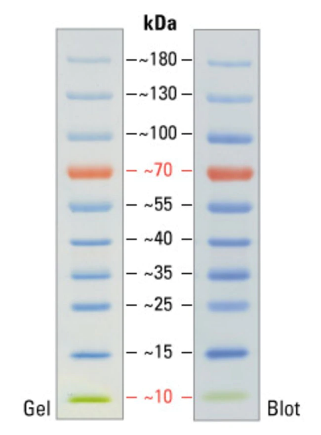


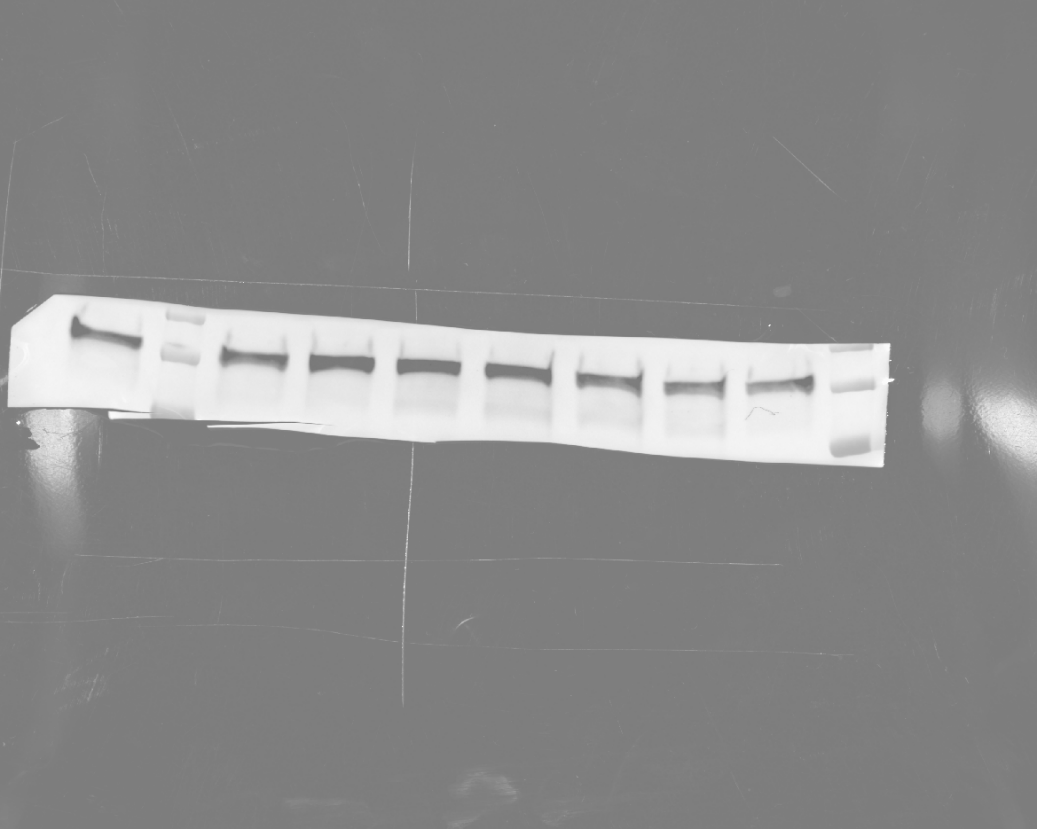


**130 kDa**


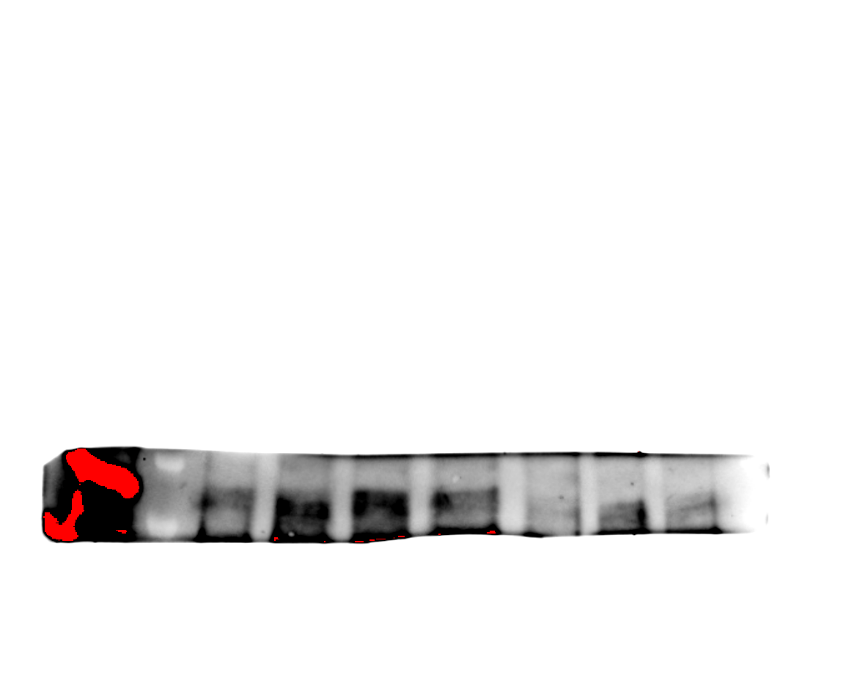

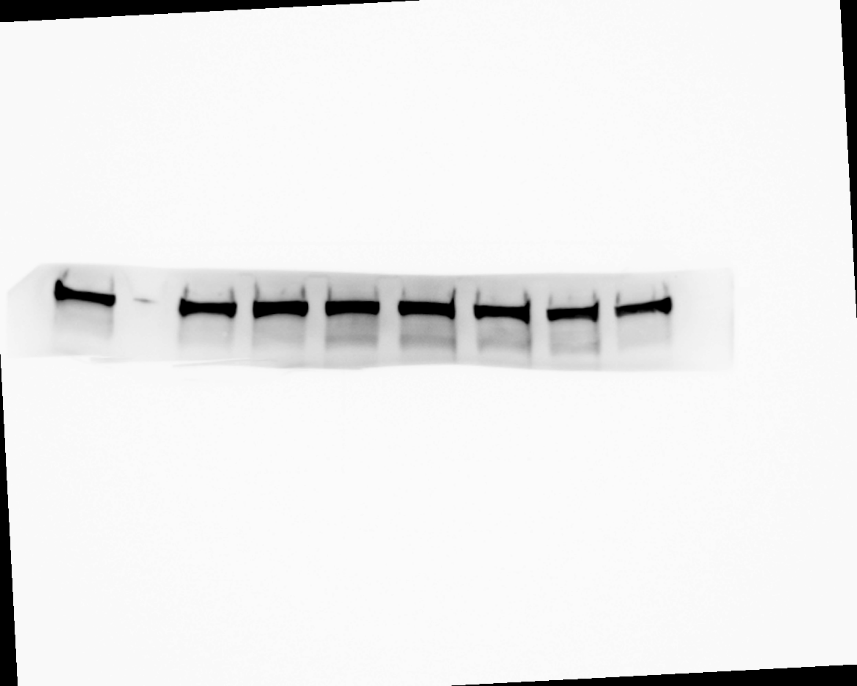

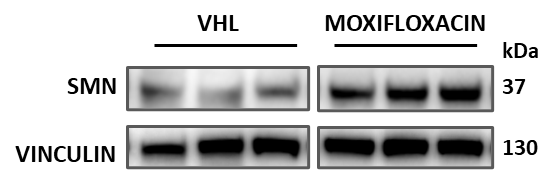

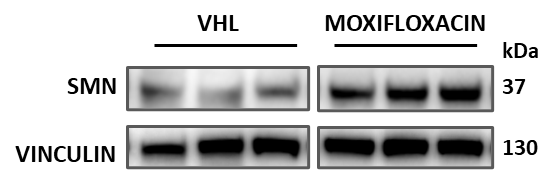

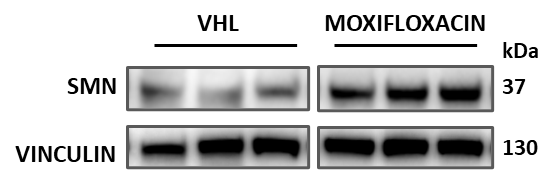

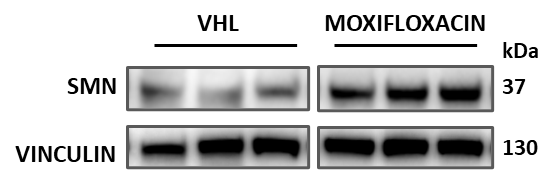


**130 kDa**

**VINCULIN**

**SMN**

**37 kDa**


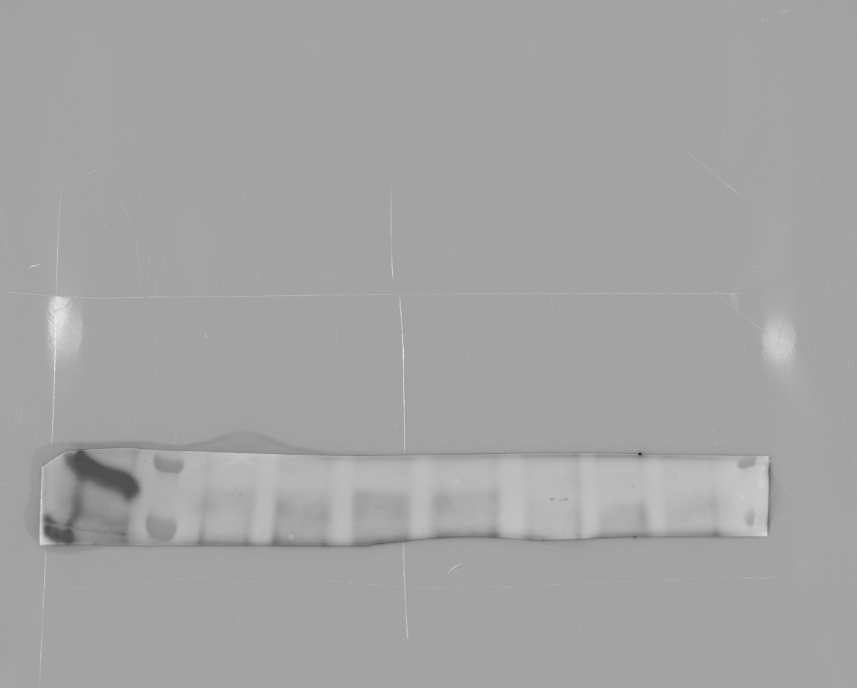

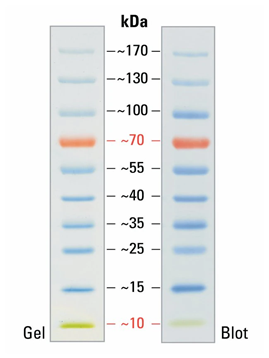

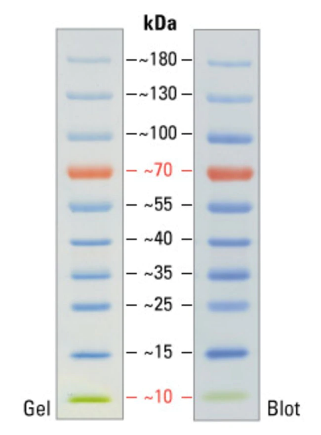


**pageruler prestained
protein ladder 26616**
