# Supplementary material for: Moxifloxacin rescues SMA phenotypes in patient-derived cells and animal model
Source: Cell Mol Life Sci. 2022 Jul 22;79(8):441. doi: 10.1007/s00018-022-04450-8 (PMC9304069; doi:10.1007/s00018-022-04450-8)
Supplement: Supplementary file 9 — Supplementary file9 (DOCX 13 kb) [file 18_2022_4450_MOESM9_ESM.docx]

**Supplementary Table 1** | Number of animals used for the weight gain analysis after treatment with moxifloxacin.

| Postnatal day | Number of animals treated with **vehicle** | Number of animals treated with **moxifloxacin** |
| --- | --- | --- |
| 2 | 21 | 9 |
| 3 | 21 | 9 |
| 4 | 21 | 9 |
| 5 | 21 | 9 |
| 6 | 18 | 9 |
| 7 | 16 | 9 |
| 8 | 17 | 9 |
| 9 | 16 | 9 |
| 10 | 15 | 9 |
| 11 | 12 | 9 |
| 12 | 12 | 9 |
